# The *MYC* Paralog-PARP1 Axis as a Potential Therapeutic Target in *MYC* Paralog-Activated Small Cell Lung Cancer

**DOI:** 10.3389/fonc.2020.565820

**Published:** 2020-10-08

**Authors:** Xing Bian, Xiaolin Wang, Qiuyan Zhang, Liying Ma, Guozhen Cao, Ao Xu, Jinhua Han, Jun Huang, Wenchu Lin

**Affiliations:** ^1^ High Magnetic Field Laboratory, Chinese Academy of Sciences, Hefei, China; ^2^ University of Science and Technology of China, Hefei, China; ^3^ Key Laboratory of High Magnetic Field and Ion Beam Physical Biology, Hefei Institutes of Physical Science, Chinese Academy of Sciences, Hefei, China; ^4^ High Magnetic Field Laboratory of Anhui Province, Hefei, China; ^5^ The CAS Key Laboratory of Innate Immunity and Chronic Disease, Innovation Center for Cell Signaling Network, School of Life Sciences, University of Science and Technology of China, Hefei, China; ^6^ The First Affiliated Hospital of University of Science and Technology of China, Division of Life Sciences and Medicine, University of Science and Technology of China, Hefei, China; ^7^ Department of Pathology, Anhui Provincial Hospital, Hefei, China; ^8^ MOE Key Laboratory for Biosystems Homeostasis & Protection and Innovation Center for Cell Signaling Network, Life Sciences Institute, Zhejiang University, Hangzhou, China

**Keywords:** small cell lung cancer, *MYC* paralog, *PARP1*, BET, DNA damage response

## Abstract

Poly (ADP-ribose) polymerase 1 (PARP1) is highly expressed in small cell lung cancer (SCLC) and has emerged as an attractive target for treatment of SCLC. However, the clinical significance of PARP1 expression in SCLC remains elusive. In this study, we showed that high PARP1 expression was associated with better overall survival (OS), and was positively correlated with the expression of *MYC* paralogs in patients with SCLC. We demonstrated that *PARP1* was transcriptionally regulated by *MYC* paralogs. Integrative analysis of multiple RNA-seq data sets indicated that DNA damage response (DDR) genes involved in the replication stress response (RSR) and homologous recombination (HR) repair pathways were highly enriched in *MYC* paralog-addicted SCLC cell models and in human SCLC specimens. Targeting the *MYC* paralog-PARP1 axis with concomitant BET and PARP inhibition resulted in synergistic effects in *MYC* paralog-activated SCLC. Our study identified a critical PARP1 regulatory pathway, and provided evidence for a rational combination treatment strategy for *MYC* paralog-activated SCLC.

## Introduction

Small cell lung cancer is a high-grade neuroendocrine carcinoma that accounts for over 250,000 annual cases worldwide (1, 2). In the past few decades, platinum-based chemotherapy with or without radiation has been the standard of care for the treatment of patients with SCLC (3). Although the initial response rate to standard treatment is high, patients with SCLC often experience relapse within one year, and the 5-year survival rate is only 7 percent (4). The SCLC treatment landscape had remained unchanged until the recent FDA approval of combined treatment using immunotherapy and chemotherapy (NCT02763579) (5). However, the development of novel therapeutic strategies to expand SCLC treatment options is a pressing need to improve clinical outcomes.

Poly (ADP-ribose) polymerase 1 (PARP1), an enzyme involved in surveillance and maintenance of genome integrity, is highly expressed in SCLC (6). Treatment with PARP inhibitors (PARPi) alone or in combination with chemotherapy has been shown to induce beneficial therapeutic effects against SCLC in preclinical and clinical studies (7–11). However, SCLC cells do not respond uniformly to PARP inhibitors (8). Therefore, investigation of the underlying mechanisms that determine therapeutic sensitivity to PARP inhibitors will allow for the targeted use of PARPi to treat SCLC.

*MYC* paralogs, including *c-MYC*, *MYCL*, and *MYCN*, play a pivotal role in tumorigenesis and tumor maintenance through regulation of a variety of cellular processes (12–15). Studies have shown that *MYC* paralogs are often exclusively amplified or overexpressed in SCLC (16, 17). Furthermore, overexpression of *c-MYC* or *MYCL* dramatically accelerated SCLC progression in genetically-engineered mouse models, which indicated that *MYC* paralogs promote oncogenesis in SCLC (18, 19). However, directly targeting *MYC* paralog has proven challenging due to the unique protein structures of the different paralogs (20). Several studies have modulated *MYC* paralog signaling through inhibition of BET, which resulted in promising anti-tumor effects against multiple cancer types, including SCLC (21–24). However, the biological significance of *MYC* paralogs in SCLC development, and the underlying mechanisms of the anti-tumor effects of BET inhibition (BETi) in SCLC, requires further characterization (25).

*MYC* paralog and *PARP1* are both amplified or overexpressed in SCLC, but the association between *MYC* paralog and *PARP1* has not been investigated in SCLC. Recent studies showed that PARP1 transcriptionally regulated *c-MYC* in quiescent cells (26), and MYCN transcriptionally regulated *PARP1* and several other DNA damage response genes in neuroendocrine prostate cancer cells (27). However, whether *MYC* paralogs activate *PARP1* in SCLC is unknown. We hypothesized that *MYC* paralogs transcriptionally activate *PARP1*, which might contribute to increased expression of PARP1 in SCLC.

In this study, we showed that patients with SCLC with high expression of *PARP1* had better prognoses than patients with low *PARP1* expression, and *PARP1* expression correlated positively with the expression of *MYC* paralogs. We also uncovered that genes related to the DDR pathway were highly enriched in *MYC* paralog-activated SCLC cells through evaluation of multiple SCLC gene expression datasets. Targeting of the *MYC* paralog-PARP1-DDR signaling pathway using the combination of BETi JQ1 and PARPi BMN673 demonstrated excellent anti-tumor efficacy in *MYC* paralog-dependent SCLC cells. In contrast, *MYC* paralog-independent SCLC cells did not respond well to this combination treatment. Finally, we showed that JQ1 and BMN673 induced synergistic effects in SCLC xenograft models and in *ex vivo* cultured PDX tumor explants. Our findings showed that inhibition of PARP and BET resulted in synergistic effects, and *MYC* paralogs were identified as possible determinants of treatment response.

## Materials and Methods

### Cell Lines and Reagents

All human small cell lung cancer cell lines were maintained in RPMI 1640 medium supplemented with 10% fetal bovine serum (FBS) and 1% penicillin/streptomycin (PS) at 37°C in a 5% CO_2_ incubator. BMN673 was purchased from Biochempartner (Shanghai, China), JQ1 was purchased from Selleck Chemical (Shanghai, China), and all drugs were dissolved in DMSO (Sigma-Aldrich, Saint Louis, MO, USA).

### SCLC Cell Line Data Processing and Unsupervised Clustering Analysis

Sequencing data (RNA-seq) from 50 SCLC cell lines, and general information for these cell lines, was downloaded from https://portals.broadinstitute.org/ccle/data. Transcriptome sequencing data from 77 human primary SCLC tumors and sample information were obtained from George et al, 2015. Sequencing data (RNA-seq) from 14 murine SCLC tumors were downloaded from GSE89660 (18). Expression data for RSR, HR repair, NHEJ pathway genes, and *MYC* paralogs were extracted, analyzed, and displayed in scatter plots or subjected to unsupervised cluster analysis and displayed in a heatmap.

### Immunohistochemistry Staining of Human SCLC Tumor Tissues

Paraffin-embedded tumor tissues were subjected to immunohistochemical staining. Four-micrometer slices were deparaffinized in xylene, then rehydrated. Then, antigen retrieval was performed for 30 min. Endogenous peroxidase activity was blocked with 30% hydrogen peroxide in methanol solution at room temperature for 30 min. Then, the slices were blocked against non-specific binding for 30 min using goat serum, and the sections were incubated with primary antibodies against PARP1 (Affinity, DF7198) and c-MYC (Abcam, ab32072) overnight at 4°C. The sections were stained using a DAB kit (Vector, SK4100). The sections were then counterstained with hematoxylin, dehydrated, and mounted. Images were captured using a Leica microscope (Leica Microsystems). All immunohistochemical staining of PARP1 and c-MYC was evaluated and quantified as the percentage of nuclear-positive cells.

### Chromatin Immunoprecipitation and PCR

Chromatin immunoprecipitation (ChIP) assay was performed as previously described (28). Cells were cross-linked using a UV cross-linker, lysed in SDS lysis buffer (1% SDS, 10 mM EDTA, and 50 mM Tris-HCl) containing complete protease-inhibitor cocktail (Roche), then incubated for 20 min on ice. The cells were sonicated for 5 min using a Sonics Vibra-Cell. A 50 μl sample of the supernatant was retained for analysis. The chromatin was incubated with magnetic beads and antibodies against c-MYC (Abcam, ab32072), MYCN (Abcam, ab16898), BRD4 (Bethyl, A301-985A50), or IgG (Cell Signaling) at room temperature for 6 h. Immunocomplexes were eluted in 1% SDS and 50 mM NaHCO_3_, and cross-links were reversed for 6 h at 65°C. The samples were digested using proteinase K for 1 h at 50°C, and DNA was extracted using a DNA isolation kit. Eluted DNA was subjected to qRT-PCR to detect enriched genomic DNA regions using the corresponding PCR primers. The primer sequences used in this study are summarized in **Supplementary Table 1**.

### Cell Viability Assay

Small cell lung cancer cells were treated with increasing concentrations of BMN673 (1 μM) or JQ1 (1 μM) alone or in combination. After 3 days of treatment, the cells were analyzed using the CellTiter-Glo luminescent assay according to the manufacturer’s instructions. The combined effect of BMN673 and JQ1 was evaluated by generating a combination index (CI) using Calcusyn software (Biosoft).

### Three-Dimensional Sphere Culture

Three-dimensional cell culture experiments were performed as previously described (29). Human SCLC cells were seeded on 96-well plates coated with 50% matrigel and 50% medium without FBS. The cells were grown in media with 2% matrigel and 2% FBS for 3 days. The media were then replaced with media containing drugs, and these media were refreshed every 3 days. Three-dimensional structures were visualized using a Leica microscope, and over 50 spherical structures were scored in the control group and each treatment group.

### Western Blot Analysis

Following drug treatment (BMN673: 1 μM, JQ1: 1 μM), the cells were lysed in RIPA buffer supplemented with protease and phosphatase inhibitors. Total proteins were quantified using a BCA Protein Assay Kit, separated using SDS-PAGE, then transferred to PVDF membranes. Antibodies against the following proteins were used for our studies: c-MYC (1:1,000, abcam ab32072), PARP1 (1:1,000, Affinity DF7198), GAPDH (1:10,000, Affinity AF7021), Rad51 (1:10,000, Abcam ab133534), PARP (1:1,000, CST 9542), γH2AX (1:1,000, CST 2577), p-CHK1 (1:1,000, Ser317, CST 12302), BRD2 (1:1,000, Proteintech 22236-1-AP), BRD3 (1:1,000, Proteintech 11859-1-AP), BRD4 (1:500, Bethyl A301-985A50), p-DNA-PKcs (1:1,000, Abcam ab124918), p-RPA32 (1:5,000, Ser4/Ser8, Novus), MYCN (1:1,000, CST 9405), MYCL (1:1,000 R&D, AF4050) and β-actin (1:10,000, Transgen HC201-02). Rabbit IgG (1:10,000, CST 7074) and mouse IgG (1:10,000, CST 7076) were used as secondary antibodies.

### Comet Assay

Comet assay was performed as previously described (30). After 48 h of drug treatment (BMN673: 1 μM, JQ1: 1 μM), the cells were harvested and subjected to neutral comet assays. Following electrophoresis, the cells were stained using SYBR gold and visualized using a Zeiss fluorescence microscope.

### Immunofluorescence Staining

Immunofluorescence staining was performed as described previously (6). Suspended cells were fixed onto glass using 1% paraformaldehyde and ethanol (70%) at the treatment endpoint. The cells were incubated with primary antibodies against γH2AX (1:500, CST 2577), RAD51 (1:500, Abcam ab133534), RPA (1:200, Abcam ab2175), and p-CHK1 (1:200, Ser317, CST 12302), then incubated with secondary antibody. The cells were visualized using a Zeiss fluorescence microscope.

### RNA Isolation and Quantitative RT-PCR Analysis

Total RNA from SCLC cells was extracted using Trizol reagent (Thermo Scientific, Rockford, IL, USA) and reverse transcribed using a cDNA Synthesis Kit (Roche, Mannheim, Germany). Quantitative PCR was performed using FastStart Essential DNA Green Master (Roche, Mannheim, Germany) on a Roche LightCycler 96 Real-Time PCR System. The primer sequences used are summarized in **Supplementary Table 2**.

### Plasmid Constructs and Stable Cell Line Generation

Lentiviral shRNA constructs to knock down *BRD2*, *BRD3*, or *BRD4* were obtained from the RNAi Consortium (Broad Institute). These shRNA sequences are summarized in **Supplementary Table 3**. In addition, DMS53 and DMS273 cells were seeded one day prior to transfection with siRNA. Effectene Transfection Reagent (QIAGEN, Hilden, Germany) was used to transfect human c-MYC and negative control siNC into the cells according to the manufacturer’s instructions. Small interfering RNA sequences are listed in **Supplementary Table 4**. For overexpression, the complementary DNA (cDNA) clone of *c-MYC* was subcloned into a pWZL-blast vector (Primer sequences are listed in **Supplementary Table 5**). SHP77 cells that stably expressed c-MYC were generated using a retroviral packaging system, and selected using blasticidin (Solarbio, China).

### Histological and Immunohistochemical Analyses

Tumor tissues were fixed in 4% formalin overnight, and then embedded in paraffin. Four-micrometer paraffin-embedded sections were stained with hematoxylin and eosin (H & E). Immunohistochemistry (IHC) was performed as previously described (31). Slides were incubated at 4°C overnight with the following primary antibodies: Ki67 (1:1,000, CST 9449), cleaved-caspase 3 (1:300, CST 9661), RAD51 (1:200, Abcam ab133534), or γH2AX (1:500, Ser139, CST 2577). Three to five random 40X fields were scored for each tumor sample. Staining intensity was quantified as the percentage of nuclear-positive cells. For the quantification of immunohistochemistry staining of human SCLC specimens, the PARP1and c-MYC staining were quantified by integrated optical density (IOD) equaled to optical density by area by using image pro plus software.

### Small Cell Lung Cancer Xenograft Mouse Models

Six-week-old athymic nude mice were subcutaneously injected with 5 x 10^6^ DMS273, H526, or H196 cells in 100 μl of PBS and 100 μl of Matrigel (BD Biosciences, Franklin, NJ, USA). Drug treatment was initiated when the tumors reached 100 mm^3^. Prior to administration, BMN673 was dissolved in 10% hydroxypropyl-β-cyclodextrin, and then administered *via* oral gavage at a dose of 0.33 mg/kg/day. In addition, JQ1 was dissolved in 0.5% methylcellulose solution, then administered *via* intraperitoneal injection at a dose of 40 mg/kg/day. Tumor sizes were measured using a caliper, and tumor volumes were determined using the following equation: tumor volume [mm^3^]=(tumor length X tumor width^2^)/2.

### Patients-Derived Xenograft (PDX) Mouse Model of Small Cell Lung Cancer and *Ex Vivo* Organ Culture of PDX Tumor Specimens

Primary tumor tissues were transplanted into the subcutaneous fat pad space of mice. *Ex vivo* organ cultures were generated by dissecting fresh tumor tissues into 1-mm^3^ blocks, which were then cultured on absorbable gelatin sponges, as previously described (32). At least three pieces of tumor tissue per sponge were examined for each treatment condition. After being allowed to recover for 24 h, the tissue blocks were treated with BMN673 or JQ1 alone or in combination for 48 h. Following treatment, the tissue blocks were fixed using paraformaldehyde and processed for histological analysis.

### Statistical Analysis

All *in vitro* analyses were repeated at least in triplicate. P-values < 0.05 were considered statistically significant. Quantitative results were analyzed using two-tailed unpaired Student’s t tests using GraphPad Prism software. For the prognostic significance study, patients were classified into two groups based on median expression levels of PARP1 in the cohort of patients with SCLC. The Kaplan–Meier product-limit method and log-rank tests were used to estimate the differences in probabilities in OS and PFS between the different groups.

## Results

### *MYC* Paralogs Positively Correlate With *PARP1* Expression, and Transcriptionally Regulate *PARP1* in SCLC Cells

**Figure 1:**
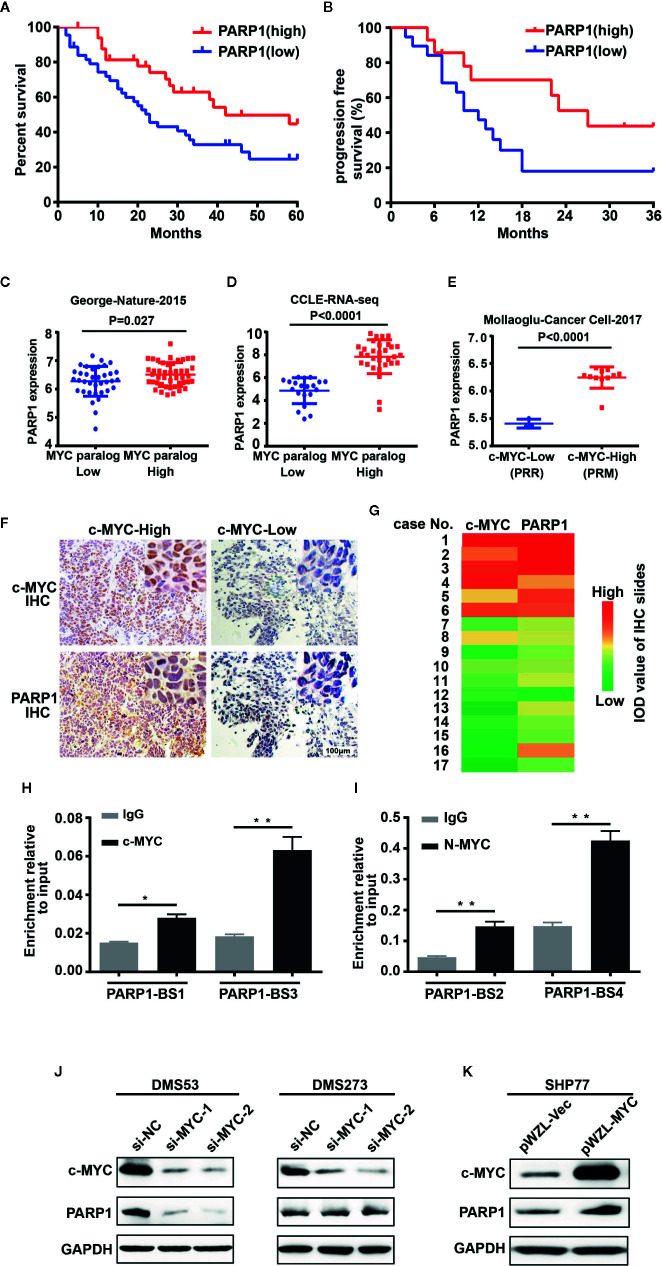
*PARP1* mRNA tightly correlates with *MYC* paralog expression and is an independent prognostic marker of survival in patients with SCLC. **(A)** Kaplan–Meier analysis of the correlation between *PARP1* expression and overall survival (OS, n = 77) and **(B)** progression-free survival (PFS, n=33). **(C–E)** Scatter plots of *PARP1* mRNA relative to expression of *MYC* paralogs in SCLC primary tumors (n=81) **(C)**, CCLE cell lines (n=50) **(D)**, murine SCLC tumors (n=14) **(E)**. **(F)** Representative images of IHC analysis of PARP1 and c-MYC in two independent cases. Scale bar, 100 μm. **(G)** Heat map showing the correlation between PARP1 and c-MYC in 17 paraffin-embedded SCLC tumor tissues. The heat map was depicted according to the IOD value of each IHC slides (red indicates c-MYC and PARP1 positive staining, green indicates negative staining). The significance analysis was performed by Student’s *t* test. **(H–I)** ChIP-qRT-PCR experiment indicating the direct binding of c-MYC and MYCN to the *PARP1* promoter in DMS273 **(H)** and H526 **(I)** cells. **(J)** Western blot analysis showing the downregulated proteins in DMS53 and DMS273 cells upon *c-MYC* knockdown. **(K)** Western blot analysis showing the upregulated proteins in SHP77 cells with ectopic *c-MYC* overexpression. GAPDH was used as a loading control. BS, binding site.

We investigated whether PARP1 could serve as a prognostic marker of SCLC by analyzing RNA-seq data from human patients with tumors (16). Patients with high levels of *PARP1* had better OS (median survival of 42 months of high PARP1 group vs 23 months of low PARP1 group, P = 0.027, log-rank test; **Figure 1A**) and progression-free survival (PFS) (median survival of 27 months of high PARP1 group vs 12 months of low PARP1 group; P = 0.061, log-rank test; **Figure 1B**) than those with low levels of *PARP1*. To gain insight into the mechanisms leading to high PARP1 expression in SCLC, we investigated *MYC* paralog expression in multiple SCLC RNA-seq data sets. An SCLC data set generated by George et al. (16) (77 human SCLC samples) showed that *PARP1* expression was higher in the group with high *MYC* paralog expression than that in the group with low *MYC* paralog expression (**Figure 1C**). In the RNA-seq data sets generated by Peifer et al. (17) (15 human SCLC samples), we noticed a strong positive correlation between PARP1 expression and *MYC* paralog expression (data not shown). Similar results were observed in the CCLE SCLC RNA-seq data set (50 human SCLC cell lines, **Figure 1D**) and the GSE89660 data set (14 murine SCLC tumors, **Figure 1E**). To further characterize the correlation between PARP1 and *MYC* paralog at the protein level in human SCLC samples, 17 human SCLC specimens were collected and subjected to immunohistochemistry analysis for c-MYC expression (**Figure 1F**). The results showed that PARP1 expression was positively associated with c-MYC expression (**Figure 1G**). These findings suggest that *PARP1* expression may be a novel prognostic marker of SCLC, and *MYC* paralog expression positively correlates with *PARP1* expression.

To provide mechanistic evidence of the potential regulation of *PARP1* by *MYC* paralog, we performed ChIP assay followed by qPCR analysis to evaluate whether *MYC* paralog transcriptionally activated *PARP1*. Four potential *MYC* paralog-binding sites in the *PARP1* promoter were analyzed. The results showed that c-MYC and MYCN directly bound to the promoter region of *PARP1* (**Figures 1H, I**). To further determine whether *c-MYC* regulated *PARP1* expression, the effects of knockdown and overexpression of *c-MYC* were evaluated in SCLC cell culture models. DMS53 and DMS273 cells were chose for the siRNA-based knockdown assay since both cell lines grow adherently and have high constitutive c-MYC expression and low c-MYC expressing SHP77 cells was used for the overexpression assay. Western blot analysis showed that knockdown of *c-MYC* using siRNA significantly suppressed the expression of PARP1 in DMS53 cells while knockdown of *c-MYC* did not significantly suppress PARP1 in DMS273 cells (**Figure 1J**). Furthermore, ectopic overexpression of *c-MYC* using a retrovirus packaging system in SHP77 cells markedly induced PARP1 expression (**Figure 1K**). These results show that c-MYC and MYCN transcriptionally regulate *PARP1* in SCLC cells.

### DNA Damage Response-Related Genes Were Enriched in *MYC* Paralog-Dependent SCLC

**Figure 2:**
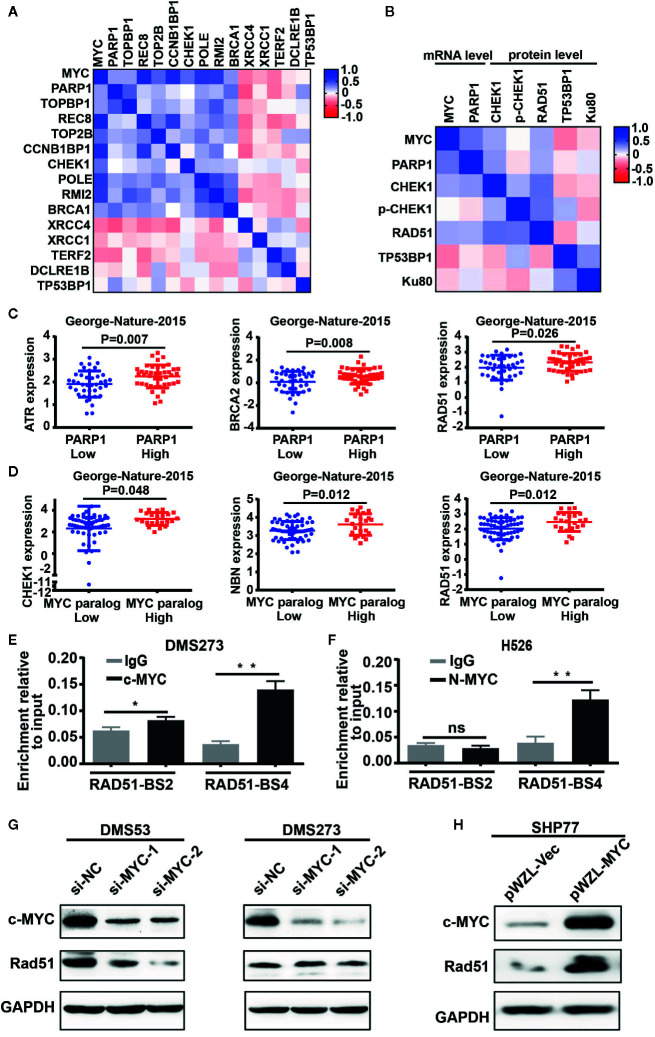
DNA damage response genes were highly enriched in SCLC cells with high expression of *MYC* paralogs or *PARP1* at mRNA levels and protein levels. **(A**, **B)** Unsupervised clustering of DDR genes and *MYC* paralogs in CCLE RNA-seq data set (n = 50) and reverse-phase protein array (RPPA) data set (**B**, n=50). **(C)** Scatter plots of *PARP1* expression relative to the expression of *ATR*, *BRCA2*, and *RAD51* in SCLC primary tumors (n=81). **(D)** Scatter plots of the expression of *MYC* paralogs relative to the expression of *CHEK1*, *NBN*, and *RAD51* in SCLC primary tumors (n=81). The significance analysis was performed by Student’s *t* test. **(E, F)** ChIP -PCR experiment indicating the binding of c-MYC and MYCN to the *RAD51* promoter in DMS273 **(E)** and H526 cells **(F)** *P < 0.05; **P < 0.01; ns, no significance. **(G)** Western blot analysis of Rad51 expression in DMS53 and DMS273 cells upon *c-MYC* knockdown. **(H)** Western blot analysis of Rad51 expression in SHP77 cells with *c-MYC* overexpression. GAPDH was used as a loading control. BS, binding site.

The above observation prompted us to further investigate the role of *MYC* paralog in the DDR pathway in SCLC. We first evaluated the CCLE RNA-seq data set for the expression of *MYC* paralogs and DDR genes. Unsupervised clustering analysis of *MYC* paralogs, *PARP1*, and other common DDR genes showed that a number of genes involved in the replication stress response and the homologous recombination repair pathways were positively correlated with the expression levels of *MYC* paralog and *PARP1* (**Figure 2A**). In contrast, genes related to non-homologous end joining (NHEJ) repair were negatively associated with *MYC* paralog expression (**Figure 2A**). Furthermore, integrative analysis of the CCLE SCLC RNA-seq data set and the RPPA data set showed that several DDR genes were highly expressed at the protein level in SCLC cells that expressed high levels of either *MYC* paralogs or *PARP1* (**Figure 2B**). In addition, several genes involved in the RSR and HR repair pathways tend to be highly expressed in SCLC specimens that exhibited high expression of *MYC* paralogs in the SCLC data set generated by George et al. (16) (**Supplementary Figure 1**). Interestingly, *ATR*, *BRCA2*, and *RAD51* were the genes most significantly associated with *PARP1* expression (**Figure 2C**), and *CHEK1*, *NBN*, and *RAD51* were the genes most significantly associated with *MYC* paralog expression (**Figure 2D**). We then wondered whether *MYC* paralogs transcriptionally regulated other DDR-related genes in addition to *PARP1* using ChIP-PCR analysis. The results showed that c-MYC and MYCN directly bound to the promoter region of *RAD51* in DMS273 (**Figure 2E**) and H526 cells (**Figure 2F**). In addition, knockdown of *c-MYC* using siRNA led to downregulation of RAD51 in DMS53, but not in DMS273 cells (**Figure 2G**). Furthermore, *c-MYC* overexpression significantly induced RAD51 expression in SHP77 cells (**Figure 2H**), which supported the observation that c-MYC transcriptionally regulated *RAD51* in SCLC cells. Our results indicate that genes involved in the RSR and HR repair pathways were necessary for maintenance of *MYC* paralog-dependent SCLC tumor growth.

### Combined PARP1 and BET Inhibition Induces Synergistic Anti-Tumor Activity in *MYC* Paralog-Dependent SCLC Cells

**Figure 3:**
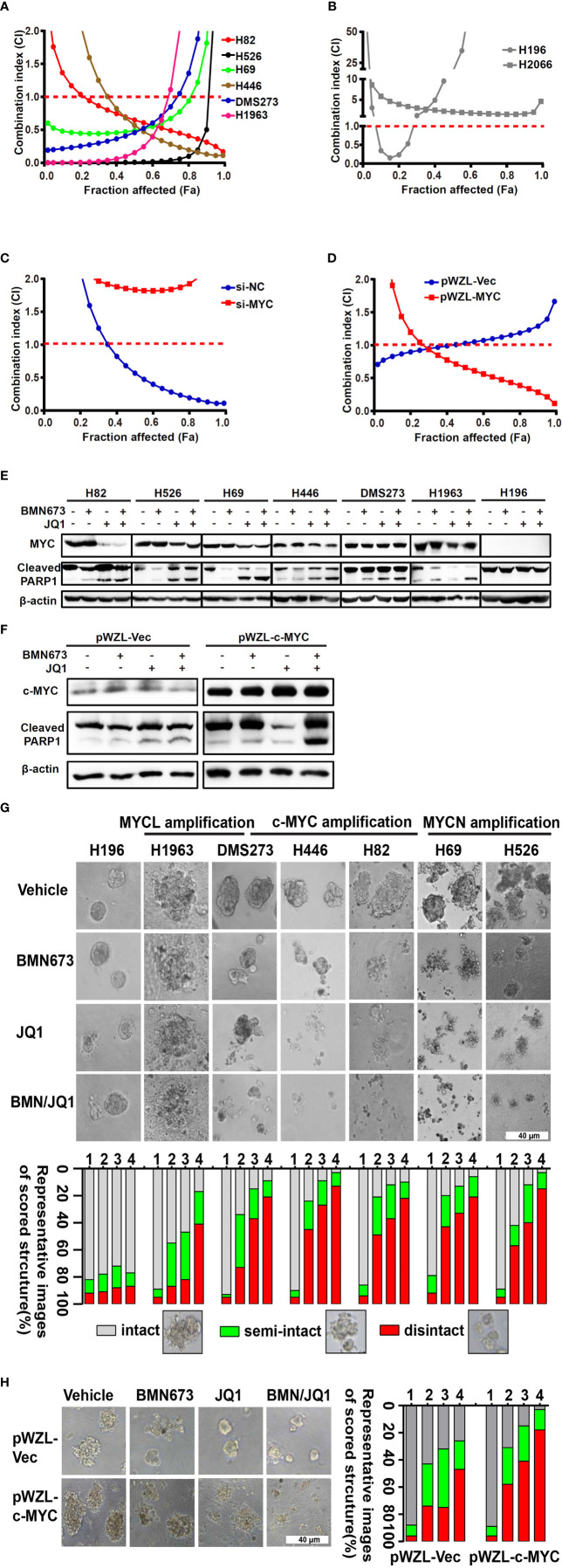
The combination effects of JQ1 and BMN673 in SCLC cells. **(A**–**D)** CellTiter-Glo Luminescent assays demonstrating the effects of JQ1 and BMN673 as single agents or in combination in *MYC* paralog-dependent **(A)**, independent **(B)** SCLC cells, DMS53 cells with c-MYC knockdown **(C)**, and SHP77 cells with *c-MYC* overexpression **(D)**. A mathematical model was applied to calculate the combination index using the CalcuSyn software program. **(E)** Western blot analysis of cleaved PARP and MYC paralogs in SCLC cells treated with BMN673 or JQ1 alone or in combination for 24 h. c-MYC for H82, H446 and DMS273, MYCN for H526 and H69, MYCL for H1963. **(F)**, Western blot analysis of cleaved PARP and c-MYC in SHP77 cells with *c-MYC* overexpression followed by BMN673 and JQ1 treatment alone or in combination for 24 h. GAPDH was used as a loading control. **(G)** Tumor sphere structures in 3D matrigel were captured under a phase-contrast microscope upon treatment of JQ1 and BMN673 as single agents or in combination for 10 to 15 days. Representative images of tumor spheres were shown in the top panel. Quantification of scored tumor sphere structures (disintegrated, semi-disintegrated, and intact) was shown in the bottom panel. Scale bar, 40 μm. **(H)** 3D matrigel assays showing the effect of JQ1 and BMN673 in SHP77 cells with or without *c-MYC* overexpression. 1, Vehicle; 2, BMN673; 3, JQ1; 4, JQ1+BMN673.

Our results highlighted the pivotal role of the *MYC* paralog-PARP1-DDR axis in SCLC. Based on these findings, we evaluated whether targeting of *MYC* paralog using JQ1 and PARP1 using BMN673 in combination could be a potential strategy for treatment of SCLC. We used a panel of SCLC cell lines with exclusive *MYC* paralog amplification and two SCLC cell lines without *MYC* paralog amplification or overexpression to assess the anti-tumor effects of JQ1 and BMN673 (**Supplementary Table 6**). The SCLC cell lines were treated with a range of concentrations of JQ1 and BMN673, alone or in combination, for 72 h. Cell viability was determined using the CellTiter-Glo luminescent assay. Combination treatment with BMN673 and JQ1 resulted in synergistic growth inhibition in all *MYC* paralog-amplified SCLC cells, but not in *MYC* paralog-non amplified SCLC cells (**Figures 3A, B**, and **Supplementary Figure 2**). Interestingly, *c-MYC* knockdown prevented the synergistic effects induced by combined treatment with BMN673 and JQ1 in DMS53 cells (**Figure 3C**), and *c-MYC* overexpression enhanced the sensitivity of SHP77 cells to combined treatment with BMN673 and JQ1 (**Figure 3D**). Western blot analysis showed that c-MYC expression was reduced by JQ1 in H446 and H82 cells but not in DMS273 cells, expressions of MYCN and MYCL were significantly suppressed by JQ1 in H526, H69 and H1963 cells (**Figure 3E**). The levels of cleaved PARP protein were significantly increased in the combined treatment group in *MYC* paralog-dependent SCLC cells compared with those in *MYC* paralog-independent SCLC cells (H196, **Figure 3E**). Furthermore, overexpression of *c-MYC* resulted in significantly increased levels of cleaved PARP protein in response to combined treatment with BMN673 and JQ1 (**Figure 3F**). It is worth noting, JQ1 treatment did not suppress c-MYC expression in SHP77 cells with *c-MYC* overexpression (**Figure 3F**). These results indicate that combined treatment with JQ1 and BMN673 induces synergistic effects, as evidenced by significant *MYC* paralog-dependent SCLC cell apoptosis.

### BMN673 and JQ1 Synergistically Inhibit Cell Growth

To evaluate the effects of combined treatment with BMN673 and JQ1 in conditions that mimic the cellular microenvironment, we generated a 3D model of SCLC cells in matrigel. Combined treatment with BMN673 and JQ1 resulted in much greater structural disintegration of 3D spheroids than treatment with JQ1 or BMN673 alone in *MYC* paralog-dependent SCLC cells (**Figure 3G**). In contrast, 3D cultures of *MYC* paralog-independent SCLC cells (H196) did not respond to BMN673 or JQ1, alone or in combination (**Figure 3G**). Furthermore, ectopic overexpression of *c-MYC* significantly increased the structural disintegration of 3D spheroids induced by combined treatment with BMN673 and JQ1 in SHP77 cells (**Figure 3H**). These results indicate that combined administration of JQ1 and BMN673 might be a potential treatment option for *MYC* paralog-dependent SCLC cells.

### BMN673 and JQ1 Cooperate to Induce DNA Damage and Attenuate the Replication Stress Response

**Figure 4:**
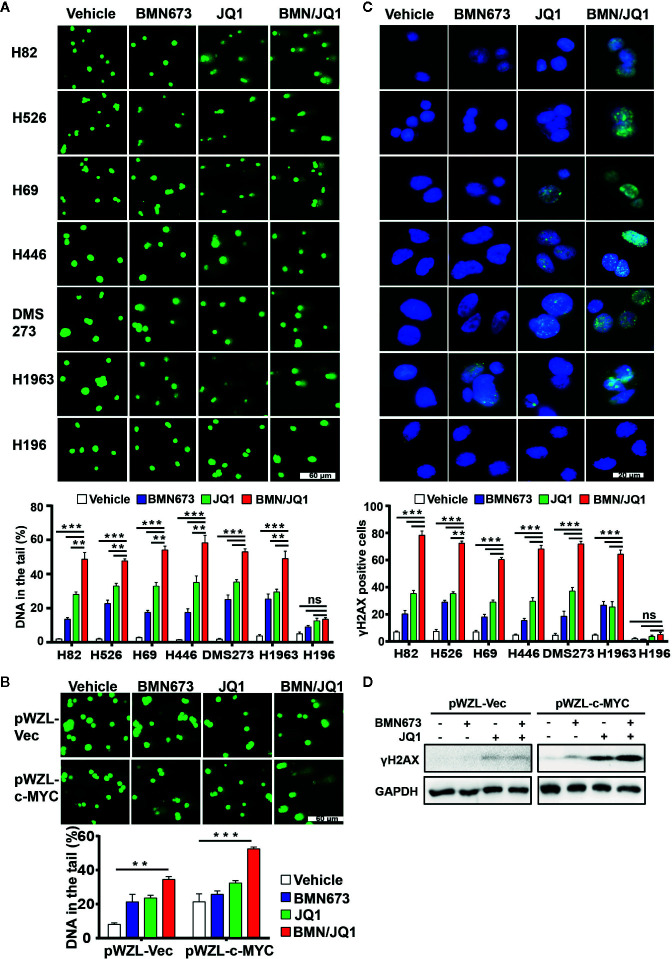
Effects of JQ1 and BMN673 on DNA damage in *MYC* paralog-dependent SCLC cells. **(A)** Comet assays detecting DNA damage in SCLC cells upon treatment with BMN673 or JQ1 alone and in combination for 48 h. **P < 0.01; ***P < 0.001. **(B)** Comet assays detecting DNA damage in SHP77 cells with or without *c-MYC* overexpression, treated with indicated drugs for 48 h. Scale bar, 60 μm. DNA in the tail was used to measure DNA damage and assessed by CASP software (CaspLab). Quantification of the amount of DNA damage was shown mean ± S.D. **P < 0.01; ***P < 0.001 (Student’s *t* test). **(C)** Representative images of immunofluorescent staining for γH2AX in SCLC cells treated with indicated drugs for 24 h, Scale bar, 20 μm. Cells with more than 5 foci were considered as positive. **P < 0.01; ***P < 0.001. **(D)** Western blot analysis of γH2AX protein levels in SHP77 cells with or without *c-MYC* overexpression treated with BMN673 or JQ1 alone or in combination for 24 h. GAPDH was used as a loading control.

To discern the molecular mechanism underlying the synergistic effects of BMN673 and JQ1 observed in SCLC cells, we evaluated the mechanisms by which BET inhibition modified RSR signaling. We first determined the extent of DNA damage using comet assay. Administration of BMN673 or JQ1 as monotherapy induced moderate DNA damage in SCLC cells, and combined treatment resulted in substantially increased DNA damage, as shown by substantial DNA tails (**Figure 4A**). In contrast, *MYC* paralog-independent SCLC cells (H196) did not show obvious DNA damage following treatment with BMN673 or JQ1, alone or in combination (**Figure 4A**). Furthermore, SHP77 cells that overexpressed *c-MYC* showed much more DNA damage than SHP77 cells that did not overexpress *c-MYC* upon the dual treatment (**Figure 4B**). In support of the comet assay results, immunofluorescence further confirmed much more elevated γH2AX foci (a marker for DNA double-strand breaks) in cells treated with the combination of BMN673 and JQ1 than those treated with either drug alone (**Figure 4C**). Cells without *MYC* paralogs amplification did not show any γH2AX foci upon BMN673 and JQ1 as monotherapy or in combination treatment (**Figure 4C**). Furthermore, western blot analysis revealed that *c-MYC* overexpression resulted in increased protein levels of γH2AX following combined treatment (**Figure 4D**). These findings indicate that combined treatment with BMN673 and JQ1 results in substantial DNA damage in *MYC* paralog-dependent SCLC cells.

**Figure 5:**
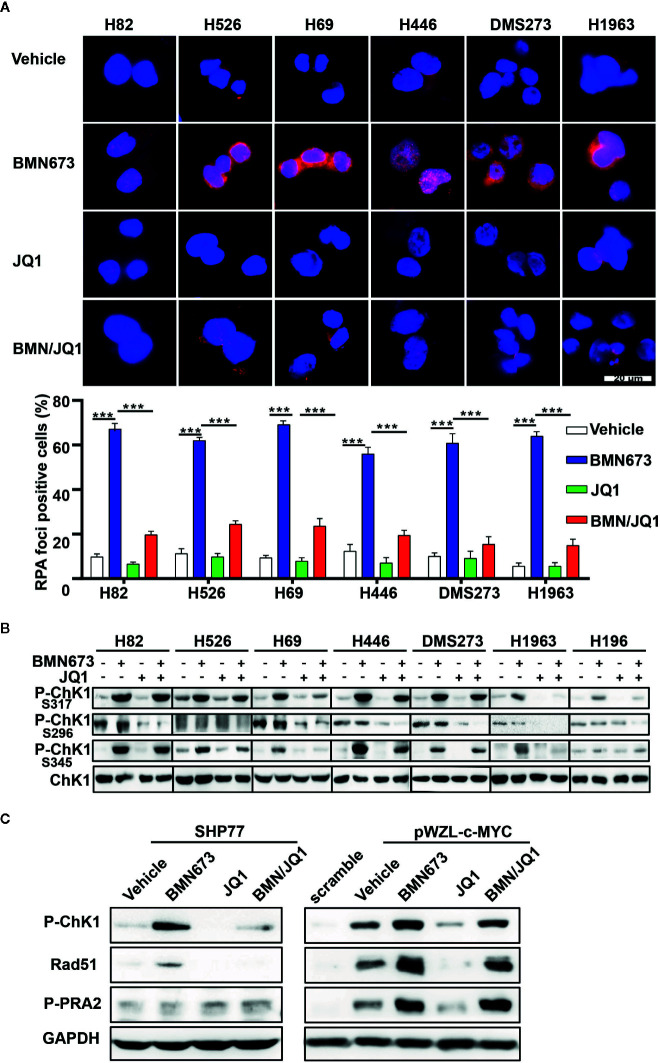
Effects of JQ1 and BMN673 on the DNA replication stress response. **(A)** Representative images of immunofluorescence staining for RPA2 in SCLC cells treated with drugs as indicated for 24 h. Scale bar, 20 μm. Quantification of RPA2 fluorescence intensities from three independent experiments was shown as mean ± S.D. ***P < 0.001. **(B)** Western blot analysis of p-CHK1 levels in *MYC* paralog-dependent and independent-SCLC cells treated with drugs as indicated for 24 h. **(C)** Western blot analysis of indicated proteins in SHP77 cells with or without *c-MYC* overexpression followed by BMN673 and JQ1 as single agents or in combination treatment for 24 h. GAPDH was used as a loading control.

Recently, PARP inhibition was shown to elicit a strong RSR in multiple cell types (33, 34). Based on this finding, we evaluated whether JQ1 could augment replication-associated DNA damage through blockade of PARPi-induced RSR. Quantitative analysis of immunofluorescence staining for the ssDNA-binding protein RPA showed that JQ1 treatment severely impaired PARPi-induced formation of RPA foci (a marker for DNA replication stress, **Figure 5A**). The ATR-CHK1 signaling axis is an essential pathway in response to replication stress (35, 36). Treatment with BMN673 resulted in substantially increased CHK1 phosphorylation in S317 and S345 sites, and this effect was reversed by treatment with JQ1, which suggested that JQ1 suppressed RSR in *MYC* paralog-dependent SCLC cells (**Figure 5B**). We also checked the phosphorylation of CHK1 in S296 site, we noticed that BMN673 did not induce strong activation of p-CHK1 in S296 site, however, JQ1 as single agent or in combination with BMN673 significantly suppressed the p-CHK1 in S296 site in most of the cell lines examined (**Figure 5B**). The *MYC* family of oncogenes plays a substantial role in induction of replication stress (37–39). We further showed that *c-MYC*-overexpressing SHP77 cells exhibited a strong RSR compared with WT SHP77 cells, as indicated by increased levels of p-CHK1, p-RPA (**Figure 5C**). Inhibition of PARP further enhanced phosphorylation of CHK1 and RPA, and JQ1 reversed this effect in *c-MYC*-overexpressing SHP77 cells (**Figure 5C** and **Supplementary Figures 3A, B**). These results were consistent with those observed in non-genetically-modified *MYC* paralog-dependent SCLC cells (**Figure 5B**). Together, these results show that BETi strongly attenuates the basal and PARPi-induced RSR in *MYC* paralog-dependent SCLC cells.

### Inhibition of BET Attenuates HR Repair in *MYC* Paralog-Dependent SCLC Cells

**Figure 6:**
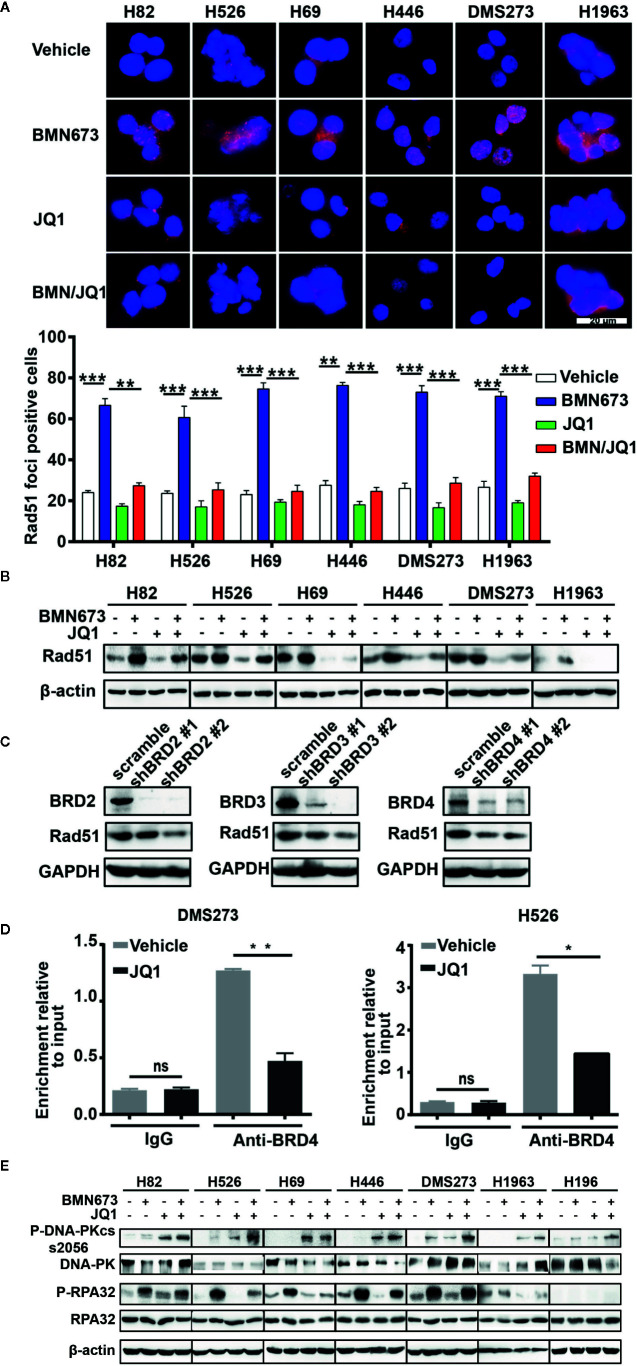
Effects of JQ1 and BMN673 on the HR repair pathway. **(A)** Representative images of Rad51 immunofluorescence staining in SCLC cells treated with drugs as indicated for 24 h. Scale bar, 20 μm. Quantification of Rad51 fluorescence intensities from three independent experiments was shown as mean ± S.D. **P < 0.01; ***P < 0.001. **(B)** Western blot analysis of Rad51 expression in SCLC cells treated with drugs as indicated for 24 h. **(C)** Western blot analysis of indicated proteins in DMS273 cell upon knockdown of *BRD2* or *BRD3* or *BRD4*. **(D)** ChIP-PCR analysis displays the decreased association of BRD4 in the promoter of *RAD51* in DMS273 and H526 cells treatment with JQ1 for 24 h. *P < 0.05; **P < 0.01; ns, no significance. **(E)** Western blot analysis of phosphorylated-DNA-PKcs and phosphorylated-RPA32 in *MYC* paralog-dependent and independent-SCLC cells treated with indicated drugs for 24 h. β-actin was used as a loading control.

PARP inhibitor activity can be modulated by HR status (40). However, previous sequencing results showed that DNA damage repair genes are rarely mutated in SCLC (16, 17). We hypothesized that JQ1 might regulate the expression of DNA damage repair genes. Real time qPCR results displayed decreased expression of *BRCA1* and *RAD51* but not *BRCA2* following JQ1 treatment (**Supplementary Figure 4**). Western blot analysis showed that JQ1 treatment resulted in dose-dependent down-regulation of Rad51 (**Supplementary Figure 5**). To further evaluate HR following drug treatment, we used immunofluorescence staining to quantify Rad51 foci (a surrogate marker of HR). Treatment with BMN673 for 24 h resulted in significantly increased formation of Rad51 foci. In contrast, combined treatment with BMN673 and JQ1 inhibited formation of Rad51 foci (**Figure 6A**), which resulted in accumulation of γH2AX foci in *MYC* paralog-dependent SCLC cells (**Figure 4C**). Furthermore, western blot analysis showed that treatment with BMN673 increased Rad51 levels, and this effect was inhibited by treatment with JQ1 (**Figure 6B**). We then investigated the mechanisms by which JQ1 inhibited formation of Rad51 foci. Messenger RNA stability assays using actinomycin D in DMS273 and H526 cells showed that JQ1 did not affect *RAD51* mRNA stability (**Supplementary Figure 6**). A recent study reported that BET proteins regulated *RAD51* in ovarian cancer cells (41), and we showed that knockdown of each BET family member (*BRD2*, *BRD3*, and *BRD4*) in DMS273 cells led to downregulation of Rad51 expression (**Figure 6C**). These results indicated that the BET family of proteins might regulate *RAD51* in SCLC cells. As BRD4 was highly expressed in SCLC cells, we wondered whether BRD4 transcriptionally regulated *RAD51* expression, we performed ChIP assay using a BRD4 antibody followed by RT-qPCR in JQ1-treated SCLC cells and control cells. The results showed that JQ1 treatment decreased the interaction between BRD4 and the promoter regions of *RAD51* (**Figure 6D**). Together, these results indicate that JQ1 reduces *RAD51* expression through suppression of the transcription of *RAD51*, but not through the destabilization of *RAD51* mRNA.

We then investigated whether knockdown of BET family proteins induced synergistic effects with BMN673 in SCLC cells. We first performed clonogenic assays, and showed that knockdown of BET proteins significantly accelerated BMN673-induced cell growth inhibition, with the most pronounced effect in *BRD4* knockdown cells (**Supplementary Figures 7A, B**). Knockdown of *BRD2* or *BRD3* resulted in modest DNA damage in BMN673-treated cells, whereas knockdown of *BRD4* resulted in substantial DNA damage following BMN673 treatment, as determined using comet assay (**Supplementary Figures 7C, E**). Furthermore, BMN673 induced significantly greater accumulation of γH2AX foci in *BRD4* knockdown cells than in *BRD2* or *BRD3* knockdown cells (**Supplementary Figures 7D, F**), which suggested that depletion of *BRD4* was sufficient to induce synergy with PARP inhibition.

Autophosphorylation of DNA-PK plays a pivotal role in NHEJ. Phosphorylation at S2056 of DNA-PKcs limits end processing during NHEJ (42, 43). Meanwhile, autophosphorylation of DNA-PKcs at S2056 site is cell cycle regulated, occurring primarily in G0/G1 cell cycle phase (44). Western blot analysis showed that JQ1 treatment alone and in combination with BMN673 led to marked increases in phosphorylation of DNA-PK at S2056 site in *MYC* paralog-dependent SCLC cells (**Figure 6E**), which suggested that JQ1 might inhibit DNA end processing and induce G1 cell cycle arrest. In accordance with these findings, JQ1 treatment markedly decreased PARPi-induced phosphorylation of RPA32 which is a DNA end-resection marker, in *MYC* paralog-dependent SCLC cells (45). However, in *MYC* paralog-independent H196 cells, we did not notice significant expression change of phosphorylated-RPA32 upon drug treatment. (**Figure 6E**) Furthermore, we showed that *c-MYC*-overexpressing SHP77 cells exhibited a strong HR, as indicated by increased levels of Rad51 (**Figure 5C**). BMN673 treatment further enhanced Rad51 expression, which was counteracted by JQ1 (**Figure 5C**), suggesting JQ1 inhibits HR in SCLC cells. Together, these results show that JQ1 treatment or knockdown of BET family members attenuates HR repair through modulation of *RAD51* expression, Rad51 foci formation, and inhibition of DNA end resection.

### Dual Inhibition of BET and PARP Is Synergistic *In Vivo*

**Figure 7:**
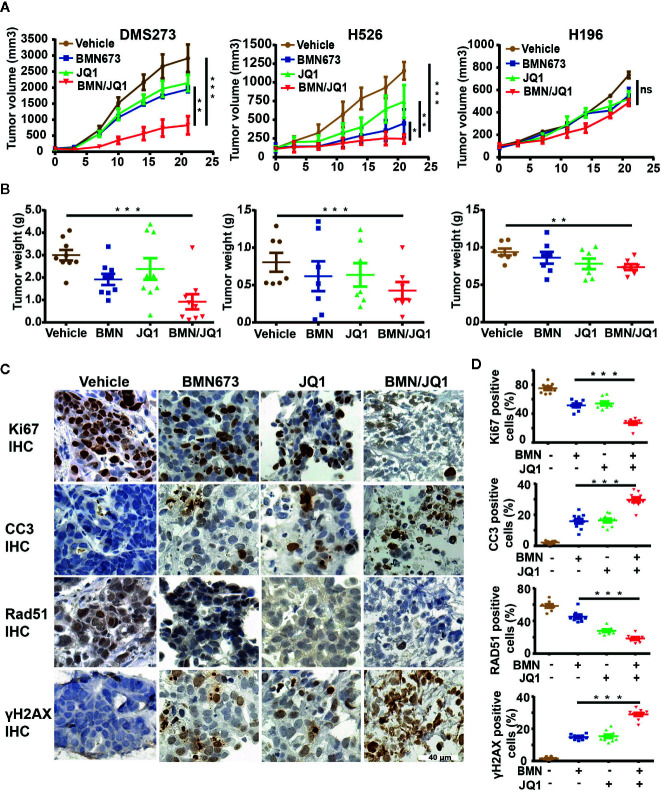
Therapeutic efficacy of JQ1 and BMN673 as single agents or in combination *in vivo*. **(A)** Tumor volume curves of DMS273 (left panel) and H526 (middle panel) and H196 (right panel) xenograft mice treated with BMN673, JQ1, or a combination of BMN673 and JQ1. Data are shown as mean ± SD (n = 6). **(B)** Tumor weights of DMS273 (left panel) and H526 (middle panel) and H196 (right panel) xenograft mice were measured after the 21 days of drug treatment. The statistic analysis was shown as mean ± S.E.M. *P < 0.05; **P < 0.01; ***P < 0.001 (Student’s *t* test). **(C)** Representative immunohistochemistry images of Ki67, cleaved-caspase3, Rad51, and γH2AX on PDX tumor explants treated with BMN673 and JQ1 alone or in combination. Scale bar, 40 μm. The statistic analysis was shown at the right panel **(D)** and indicated as mean ± S.E.M. *P < 0.05; **P < 0.01; ***P < 0.001 (Student’s *t* test).

The demonstrated synergistic effects induced by BET inhibition and PARP inhibition *in vitro* led to the evaluation of the therapeutic efficacy of BMN673 and JQ1 as monotherapies or in combination in SCLC xenograft models. SCLC cell lines, including DMS273 (*c-MYC* and *MYCL* amplification), H526 (*MYCN* amplification), and H196 (no *MYC* paralog amplification or overexpression) were selected for evaluation *in vivo*. SCLC cells were injected into the subcutaneous tissue of nude mice, and tumors were allowed to grow to about 100 mm^3^ prior to drug administration. After three weeks treatment, BMN673 or JQ1 alone attenuated tumor growth (**Figure 7A**, tumor volume reduced by 1.5-fold and 1.36-fold, respectively, for DMS273, tumor volume reduced by 1.86-fold and 1.52-fold, respectively, for H526) and reduced tumor weight (**Figure 7B**). However, combination treatment with BMN673 and JQ1 inhibited tumor growth and reduced tumor weight to a much greater extent than either drug alone (**Figures 7A, B**, tumor volumes reduced by 3.53-fold and 2.85-fold, respectively). In contrast, neither BMN673 nor JQ1, alone or in combination, inhibited tumor growth in H196 xenografts, which indicated that combined BMN673-JQ1 treatment was not effective against *MYC* paralog-independent SCLC *in vivo* (**Figures 7A, B**). Notably, all treatments were well tolerated, and did not induce significant body weight loss (**Supplementary Figures 8A–C**). To evaluate the effects of BMN673 and JQ1 on proliferation, apoptosis, and DDR, tumor tissues were analyzed using immunohistochemical staining. The results showed substantially decreased numbers of Ki67-positive tumor cells and significantly increased numbers of cleaved-caspase 3-positive cells in the group treated with BMN673 and JQ1 compared to those in the vehicle group and the monotherapy groups (**Supplementary Figures 9A, B**). Tumors in the group administered the combination of BMN673 and JQ1 showed the greatest upregulation of γH2AX expression and downregulation of Rad51 expression (**Supplementary Figures 9A, B**), which further demonstrated that this combination therapy compromised HR repair activity and enhanced DNA damage.

To further investigate the treatment effects of BMN673 and JQ1 *in vivo*, a PDX model of SCLC (c-MYC-overexpression) that maintained the pathological characteristics of the human primary tumor sample was established in our laboratory (**Supplementary Figure 10A**). We then prepared *ex vivo* organ cultures to evaluate the therapeutic effects of combination treatment using BMN673 and JQ1. Histological analysis demonstrated that the architecture and cellularity of the explants were very similar to those in the primary tumor (**Supplementary Figure 10B**). Immunohistochemistry analysis showed that combination treatment resulted in substantially reduced ki67 expression and enhanced cleaved-caspase 3 expression compared with those observed in groups treated with BMN673 or JQ1 monotherapy (**Figures 7C, D**). Furthermore, the expression of the HR repair protein Rad51 was markedly attenuated, and γH2AX expression levels were significantly increased, in explants treated with the combination of BMN673 and JQ1 (**Figures 7C, D**). Collectively, our *in vivo* study suggested that combined administration of BMN673 and JQ1 induced synergistic anti-tumor effects against *MYC* paralog-dependent SCLC xenografts and PDX explants.

## Discussion

**Figure 8:**
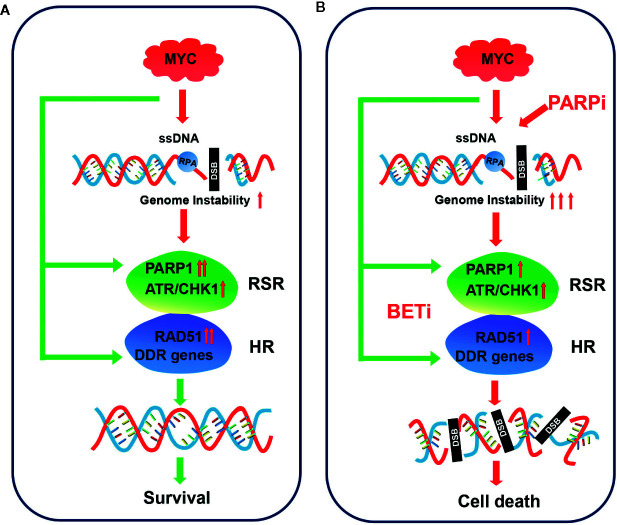
Model for targeting *MYC* paralog-PARP1 axis in *MYC* paralog-dependent SCLC cells. **(A)** Genome instability is common in *MYC* paralog-activated SCLC cells. DDR genes, including PARP1 and RAD51, were highly expressed to maintain genome stability for cell survival in this context. **(B)** Combining PARP inhibition (PARPi) with BET inhibition (BETi) yields synergistic anti-tumor effects in *MYC* paralog-activated SCLC cells. BETi treatment causes defects in the replication stress response (RSR) and homologous recombination (HR) competency. The combination of PARPi and BETi leads to synthetic lethality due to unrepaired DSBs.

Small cell lung cancer is among the most aggressive types of cancer, and therapeutic options are limited. In the current study, we showed that PARP1, a promising therapeutic target in SCLC, might be a potential prognostic marker. Integrative analysis of several gene expression data sets revealed that genes involved in the RSR and HR repair pathways, including *PARP1*, were strongly associated with the expression of *MYC* paralogs (**Figure 8**). We further showed that c-MYC and MYCN transcriptionally activated *PARP1* in SCLC cells. These findings highlighted a novel mechanism of induction of high expression of *PARP1* in a subset of SCLC. We also showed that the RSR and HR repair pathways might be potential therapeutic targets for the treatment of *MYC* paralog-dependent SCLC. Furthermore, targeting the *MYC* paralog-PARP1-DDR axis *via* concurrent inhibition of BET and PARP1 resulted in synergistic anti-tumor activity *in vitro* and *in vivo* against *MYC* paralog-driven SCLC cells (**Figure 8**), but not against *MYC* paralog-independent SCLC cells. Mechanistic investigations confirmed that BETi contributed to PARPi sensitivity through multiple mechanisms. Inhibition of BET resulted in (1) defects in the cellular response to PARPi-induced DNA replication stress, (2) impaired HR repair capacity *via* interference with DNA end resection and RAD51 function, and (3) limited end processing during NHEJ repair through induction of phosphorylation of DNA-PKcs. Finally, we showed that *MYC* paralog might be an indicator of treatment response to combination therapy with JQ1 and BMN673 in patients with SCLC.

The underlying mechanisms that cause a high expression of PARP1 in SCLC have not been characterized. We, for the first time, showed that c-MYC and MYCN directly targeted *PARP1*, and transcriptionally regulated its expression in SCLC cells. Whether MYCL directly targets *PARP1* in SCLC cells requires further study. We also demonstrated that c-MYC directly regulated *RAD51* expression using *c-MYC* overexpression or knockdown models and ChIP-PCR assays. These results agreed with those from studies that reported an association between c-MYC and the *RAD51* promoter in other cell types (46, 47). Given that a number of genes required for the RSR and HR repair pathways are highly expressed in *MYC* paralog-activated cells, we speculated that *MYC* paralogs might also regulate other DDR genes in SCLC cells. Although PARP1 expression was recently shown to be associated with poor prognosis in patients with neuroblastoma (33), we found that high PARP1 expression was associated with high OS and PFS in patients with SCLC. This discrepancy may be clarified as SCLC is usually detected in a late disease stage and PARP1 mostly acts at later points of cancer development. Meanwhile, a previous related study also indicated the favorable role of PARP1 in cancer development and the fact is that strong PARP activation may result in depletion of NAD+ and ATP which in turn cause cell death (48). These results agreed with those in a previous study that reported high nuclear PARP1 expression resulted in improved survival in patients with PDA (49). Therefore, the data present in this study can only serve as a prognosis outlook, further mechanistic investigation of PARP1 biology in SCLC is required.

PARPi induces synthetic lethality in tumors with *BRCA1/2* deficiency. However, the *BRCA1/2* mutations are rarely reported in SCLC (17, 50), which might limit PARPi efficacy. In our investigations, BMN673 treatment strongly induced DDR activation and caused modest DNA damage in *MYC* paralog-dependent SCLC (**Figures 4A, C**), which might be due to a high competency of RSR and HR in these cells. A recent study identified c-MYC expression as a predictive biomarker of CHK1 inhibitor activity in SCLC (51). Another study showed that defective HR repair or *MYC* family oncogene amplification predisposed a SCLC CDX model to a combination treatment of PARP inhibitor and WEE1 inhibitor (52). Also, *c-MYC* and *MYCN* amplification is a predictive biomarker for PARP inhibitor sensitivity in glioblastoma (53). Most importantly, we and others recently demonstrated that inhibiting RSR pathway is effective in treatment of SCLC (54, 55). Together, these studies demonstrated that targeting one or more DDR proteins is a desirable strategy for the treatment of a subset of SCLC. We further showed that *MYC* paralog-activated SCLC cells might depend on DDR signaling for cell survival. Targeting the *MYC* paralog-PARP1-DDR signaling pathway could allow for increased efficacy of PARPi.

Although we showed that c-MYC strongly induced *PARP1* and *RAD51* expression, in DMS273 cells which harbor both *c-MYC* and *MYCL* amplification, knockdown of *c-MYC* did not significantly repress PARP1 and Rad51 expression, we speculate this discrepancy may result from the transcription regulation of *PARP1* and *RAD51* by MYCL in DMS273 cells. Therefore, the effects of *MYCN* or *MYCL* on SCLC cells require further investigations. In our study, the expression of c-MYC in DMS273 cells and SHP77 cells with *c-MYC* overexpression was not altered following treatment with JQ1 (**Supplementary Figure 5**). This finding was consistent with those from a previous study that showed that JQ1 treatment did not reduce c-MYC activity (23, 56). In contrast, MYCN expression was significantly reduced by JQ1 in a dose-dependent manner in SCLC (**Supplementary Figure 5**). Whether *MYC* paralogs are required for the anti-proliferative effect of JQ1 in SCLC requires further study.

A recent finding identified a novel role for the BET family member BRD4 in the regulation of the RSR pathway through interaction with the pre-replication factor CDC6 (57). Furthermore, recent studies have shown that BRD4 inhibitors reduced HR competence through the downregulation of DDR-related gene expression (41, 58). Indeed, previous studies have shown that JQ1 can be used effectively to treat SCLC, but the underlying mechanisms of this anti-tumor activity are not well understood. Our study showed that JQ1 induced DNA damage and reduced HR repair and RSR efficiency in *MYC* paralog-dependent SCLC cells. These findings were consistent with those from a recent study that showed that BRD4 inhibition impaired HR *via* interfering with DNA end resection (58). In contrast to their proposed mechanism, we found that *BRD4* expression was not positively correlated with *RBBP8* (CtIP, a key component of DNA end resection) expression at the mRNA level (**Supplementary Figures 11A, B**). Instead, we found that JQ1 induced a marked increase in phosphorylation of DNA-PKcs at S2056. We hypothesized that JQ1-induced HR deficiency might have been due to DNA-PKcs-mediated attenuation of DNA end resection. A recent study indicated that BET inhibition hampered NHEJ activity through inhibition of ku80 (59). Given that phosphorylation of DNA-PKcs at S2056 enhances NHEJ, it would be interesting to address whether JQ1 promotes NHEJ in SCLC.

Although the FDA recently approved chemotherapy plus immunotherapy as a first-line treatment for patients with SCLC, the results from clinical trials showed only a 2-month increase in overall survival in patients treated with chemotherapy in combination with anti-PD-L1 therapy (5). Therefore, identification of new combination treatment strategies to improve clinical outcomes of patients with SCLC is an urgent need. We showed that targeting epigenetic factors *via* BET inhibition potentiated sensitivity of *MYC* paralog-dependent SCLC to the PARP inhibitor BMN673 *in vitro* and *in vivo*. Further clinical trials are needed to evaluate the efficacy of combination therapies that inhibit BET and PARP, as this strategy has great potential to improve the prognoses of patients with SCLC with *MYC* paralog amplification or overexpression.

## Data Availability Statement

Publicly available datasets were analyzed in this study, these data can be found here: https://portals.broadinstitute.org/ccle/data, George et al, 2015 (16) and NCBI Gene Expression Omnibus (GSE89660).

## Ethics Statement

The studies involving human participants were reviewed and approved by Ethics Committee of Hefei Institutes of Physical Science, Chinese Academy of Sciences. The patients/participants provided their written informed consent to participate in this study.

## Author Contributions

WL and XB conceived and designed the study, and wrote the manuscript. XB and XW performed the experiments and acquired the data. QZ, LM, and JHH performed flow cytometry analysis. AX and GC analyzed and interpreted the data. JH provided plasmids. All authors contributed to the article and approved the submitted version.

## Funding

This study is supported by National Natural Science Foundation of China (Grant Numbers: 81972191 and 81672647), Science and Technology Service Network Initiative of Chinese Academy of Sciences (Grant Number: KFJ-STS-SCYD-010), Science and Technology Major Project of Anhui Province (Grant Number: 18030801140), Key program of 13^th^ five-year plan of CASHIPS (Grant Number: KP-2017-26), and the 100-Talent Program of Chinese Academy of Sciences.

## Conflict of Interest

The authors declare that the research was conducted in the absence of any commercial or financial relationships that could be construed as a potential conflict of interest. 
