# Corrigendum: The *MYC* paralog-PARP1 axis as a potential therapeutic target in *MYC* paralog-activated small cell lung cancer

**DOI:** 10.3389/fonc.2023.1192526

**Published:** 2023-04-12

**Authors:** Xing Bian, Xiaolin Wang, Qiuyan Zhang, Liying Ma, Guozhen Cao, Ao Xu, Jinhua Han, Jun Huang, Wenchu Lin

**Affiliations:** ^1^ High Magnetic Field Laboratory, Chinese Academy of Sciences, Hefei, China; ^2^ University of Science and Technology of China, Hefei, China; ^3^ Key Laboratory of High Magnetic Field and Ion Beam Physical Biology, Hefei Institutes of Physical Science, Chinese Academy of Sciences, Hefei, China; ^4^ High Magnetic Field Laboratory of Anhui Province, Hefei, China; ^5^ The CAS Key Laboratory of Innate Immunity and Chronic Disease, Innovation Center for Cell Signaling Network, School of Life Sciences, University of Science and Technology of China, Hefei, China; ^6^ The First Affiliated Hospital of University of Science and Technology of China, Division of Life Sciences and Medicine, University of Science and Technology of China, Hefei, China; ^7^ Department of Pathology, Anhui Provincial Hospital, Hefei, China; ^8^ MOE Key Laboratory for Biosystems Homeostasis & Protection and Innovation Center for Cell Signaling Network, Life Sciences Institute, Zhejiang University, Hangzhou, China

**Keywords:** small cell lung cancer, MYC paralog, PARP1, BET, DNA damage response

**Error in Figure/Table**

In the published article, there was an error in **Figure 3E** as published. The representative picture of western blot bands of cleaved PARP1 of H446 cells were presented incorrectly. The corrected **Figure 3E** and its caption appear below.

**Figure 3:**
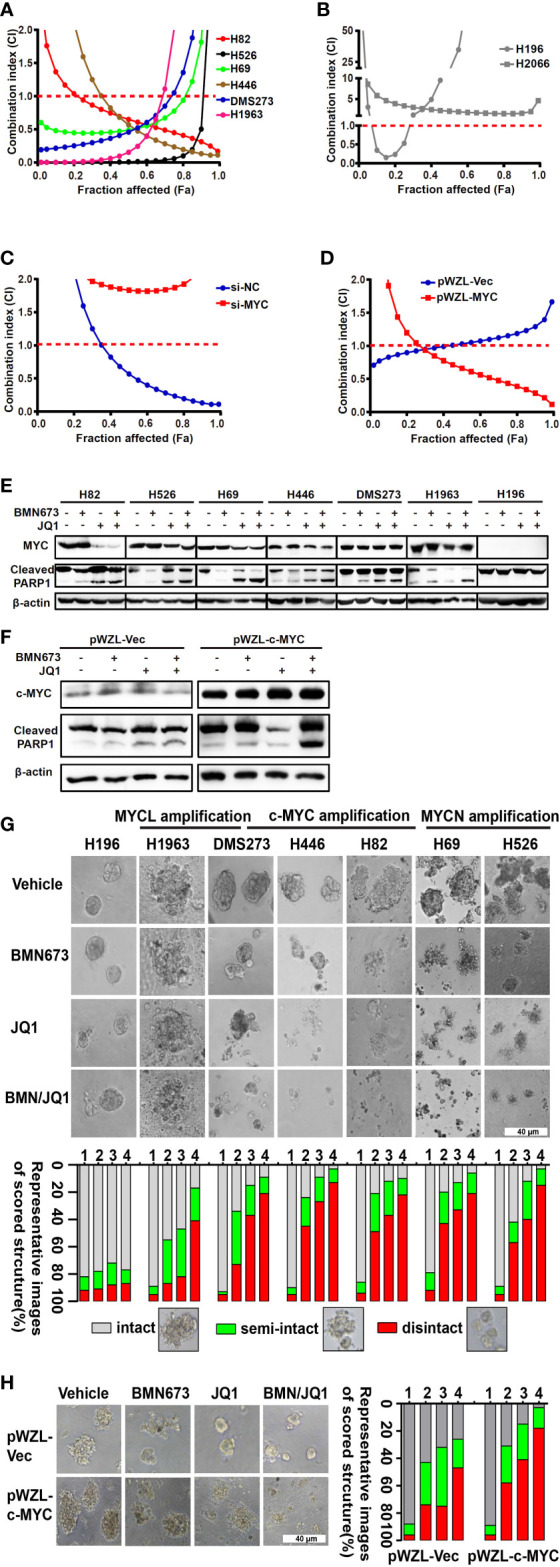
The combination effects of JQ1 and BMN673 in SCLC cells. **(A–D)** CellTiter-Glo Luminescent assays demonstrating the effects of JQ1 and BMN673 as single agents or in combination in *MYC* paralog-dependent **(A)**, independent **(B)** SCLC cells, DMS53 cells with c-MYC knockdown **(C)**, and SHP77 cells with *c-MYC* overexpression **(D)**. A mathematical model was applied to calculate the combination index using the CalcuSyn software program. **(E)** Western blot analysis of cleaved PARP and MYC paralogs in SCLC cells treated with BMN673 or JQ1 alone or in combination for 24 h. c-MYC for H82, H446 and DMS273, MYCN for H526 and H69, MYCL for H1963. **(F)**, Western blot analysis of cleaved PARP and c-MYC in SHP77 cells with *c-MYC* overexpression followed by BMN673 and JQ1 treatment alone or in combination for 24 h. GAPDH was used as a loading control. **(G)** Tumor sphere structures in 3D matrigel were captured under a phase-contrast microscope upon treatment of JQ1 and BMN673 as single agents or in combination for 10 to 15 days. Representative images of tumor spheres were shown in the top panel. Quantification of scored tumor sphere structures (disintegrated, semi-disintegrated, and intact) was shown in the bottom panel. Scale bar, 40 μm. **(H)** 3D matrigel assays showing the effect of JQ1 and BMN673 in SHP77 cells with or without *c-MYC* overexpression. 1, Vehicle; 2, BMN673; 3, JQ1; 4, JQ1+BMN673.

The authors apologize for this error and state that this does not change the scientific conclusions of the article in any way. The original article has been updated.

