# Supplementary material for: The MYC Paralog-PARP1 Axis as a Potential Therapeutic Target in MYC Paralog-Activated Small Cell Lung Cancer
Source: Front Oncol. 2020 Oct 8;10:565820. doi: 10.3389/fonc.2020.565820 (PMC7578565; doi:10.3389/fonc.2020.565820)
Supplement: Supplementary file 1 [file DataSheet_1.pdf]

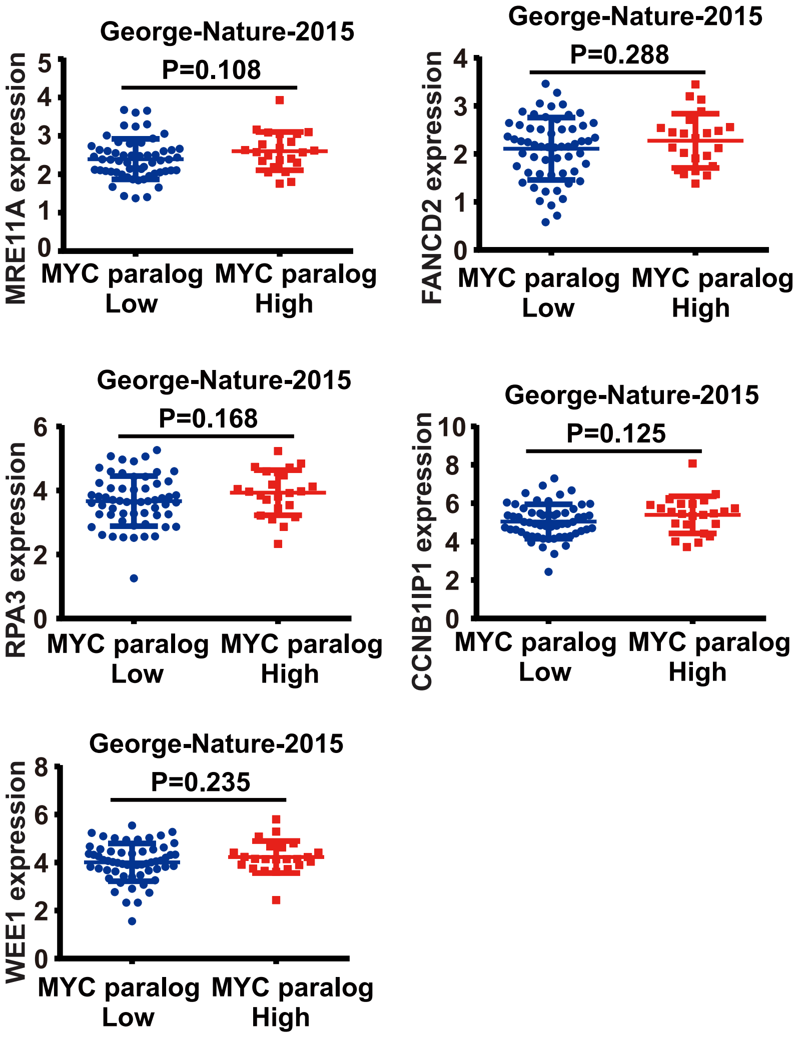
George-Nature-2015
P=0.108
MRE11A expression
0
1
2
3
4
5
MYC paralog Low
MYC paralog High
George-Nature-2015
P=0.288
FANCD2 expression
0
1
2
3
4
MYC paralog Low
MYC paralog High
George-Nature-2015
P=0.168
RPA3 expression
0
2
4
6
MYC paralog Low
MYC paralog High
George-Nature-2015
P=0.125
CCNB1IP1 expression
0
2
4
6
8
10
MYC paralog Low
MYC paralog High
George-Nature-2015
P=0.235
WEE1 expression
0
2
4
6
8
MYC paralog Low
MYC paralog High

Figure S1.

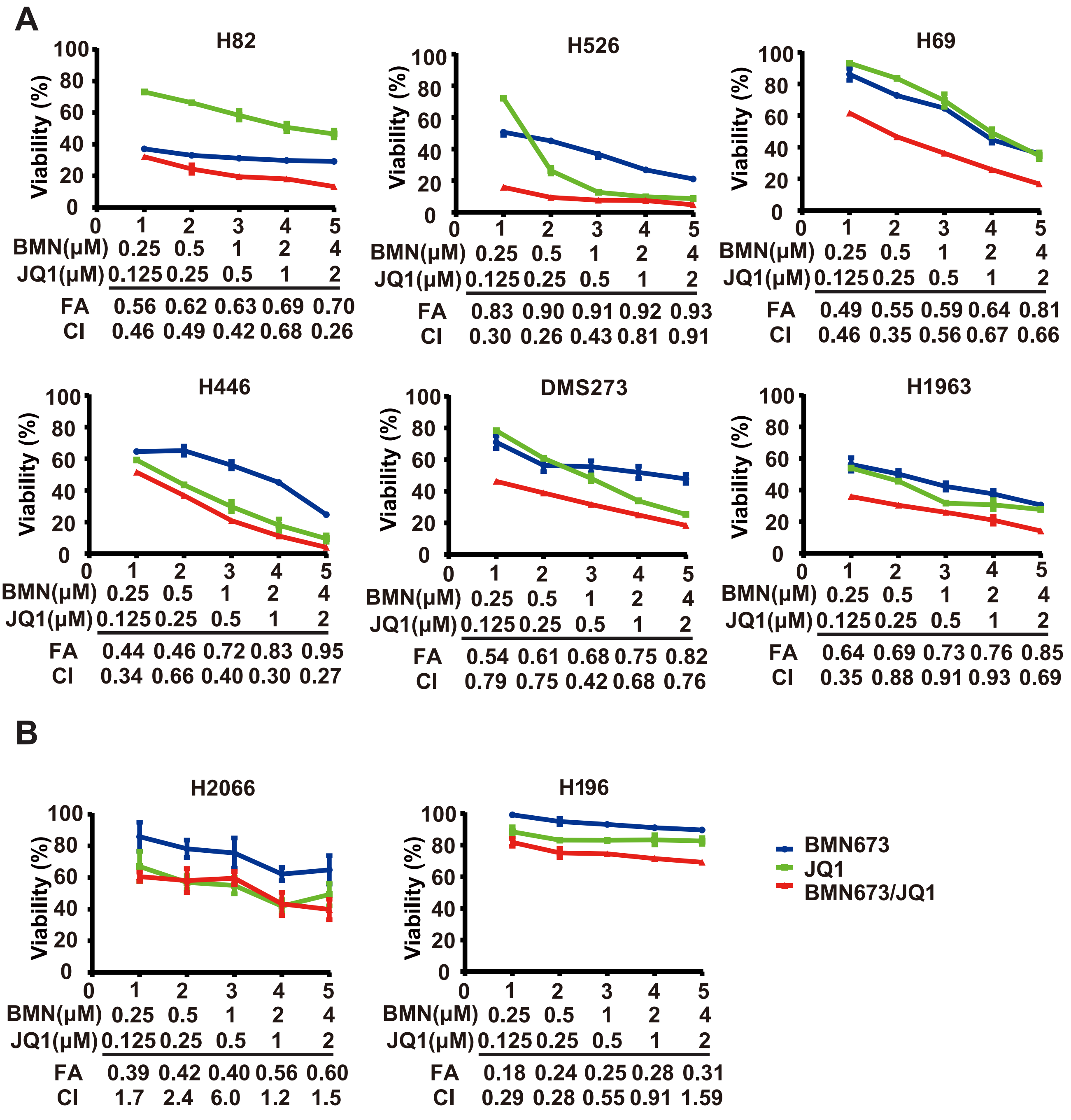
A
H82
Viability (%)
BMN(μM) 0.25 0.5 1 2 4
JQ1(μM) 0.125 0.25 0.5 1 2
FA 0.56 0.62 0.63 0.69 0.70
CI 0.46 0.49 0.42 0.68 0.26
H526
BMN(μM) 0.25 0.5 1 2 4
JQ1(μM) 0.125 0.25 0.5 1 2
FA 0.83 0.90 0.91 0.92 0.93
CI 0.30 0.26 0.43 0.81 0.91
H69
BMN(μM) 0.25 0.5 1 2 4
JQ1(μM) 0.125 0.25 0.5 1 2
FA 0.49 0.55 0.59 0.64 0.81
CI 0.46 0.35 0.56 0.67 0.66
H446
BMN(μM) 0.25 0.5 1 2 4
JQ1(μM) 0.125 0.25 0.5 1 2
FA 0.44 0.46 0.72 0.83 0.95
CI 0.34 0.66 0.40 0.30 0.27
DMS273
BMN(μM) 0.25 0.5 1 2 4
JQ1(μM) 0.125 0.25 0.5 1 2
FA 0.54 0.61 0.68 0.75 0.82
CI 0.79 0.75 0.42 0.68 0.76
H1963
BMN(μM) 0.25 0.5 1 2 4
JQ1(μM) 0.125 0.25 0.5 1 2
FA 0.64 0.69 0.73 0.76 0.85
CI 0.35 0.88 0.91 0.93 0.69
B
H2066
BMN(μM) 0.25 0.5 1 2 4
JQ1(μM) 0.125 0.25 0.5 1 2
FA 0.39 0.42 0.40 0.56 0.60
CI 1.7 2.4 6.0 1.2 1.5
H196
BMN(μM) 0.25 0.5 1 2 4
JQ1(μM) 0.125 0.25 0.5 1 2
FA 0.18 0.24 0.25 0.28 0.31
CI 0.29 0.28 0.55 0.91 1.59
BMN673
JQ1
BMN673/JQ1

Figure S2.

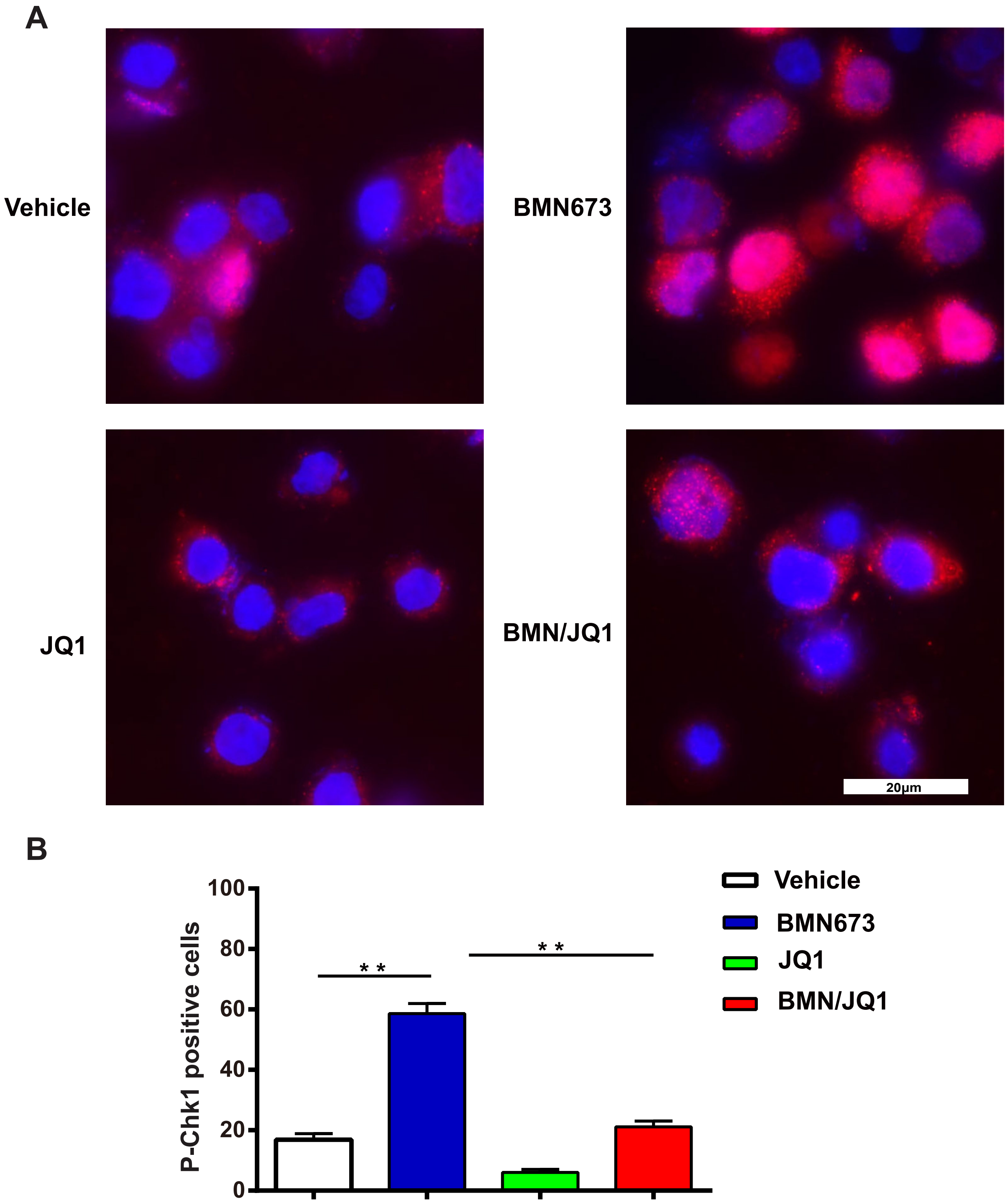
A
Vehicle
BMN673
JQ1
BMN/JQ1
20μm
B
P-Chk1 positive cells
100
80
60
40
20
0
* *
* *
Vehicle
BMN673
JQ1
BMN/JQ1

Figure S3.

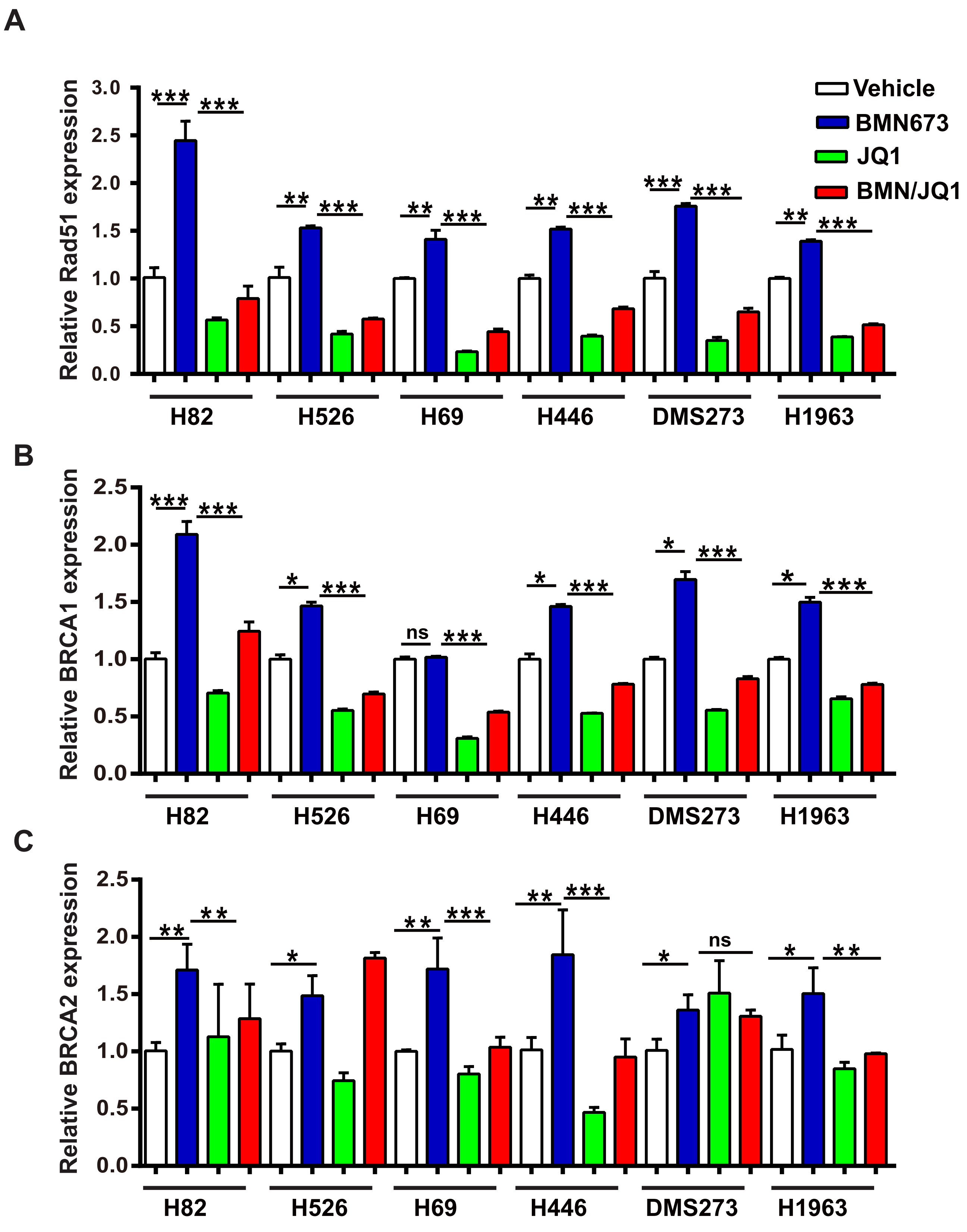
A
Relative Rad51 expression
3.0
2.5
2.0
1.5
1.0
0.5
0.0
Vehicle
BMN673
JQ1
BMN/JQ1
H82
H526
H69
H446
DMS273
H1963
B
Relative BRCA1 expression
2.5
2.0
1.5
1.0
0.5
0.0
ns
H82
H526
H69
H446
DMS273
H1963
C
Relative BRCA2 expression
2.5
2.0
1.5
1.0
0.5
0.0
ns
H82
H526
H69
H446
DMS273
H1963

Figure S4.

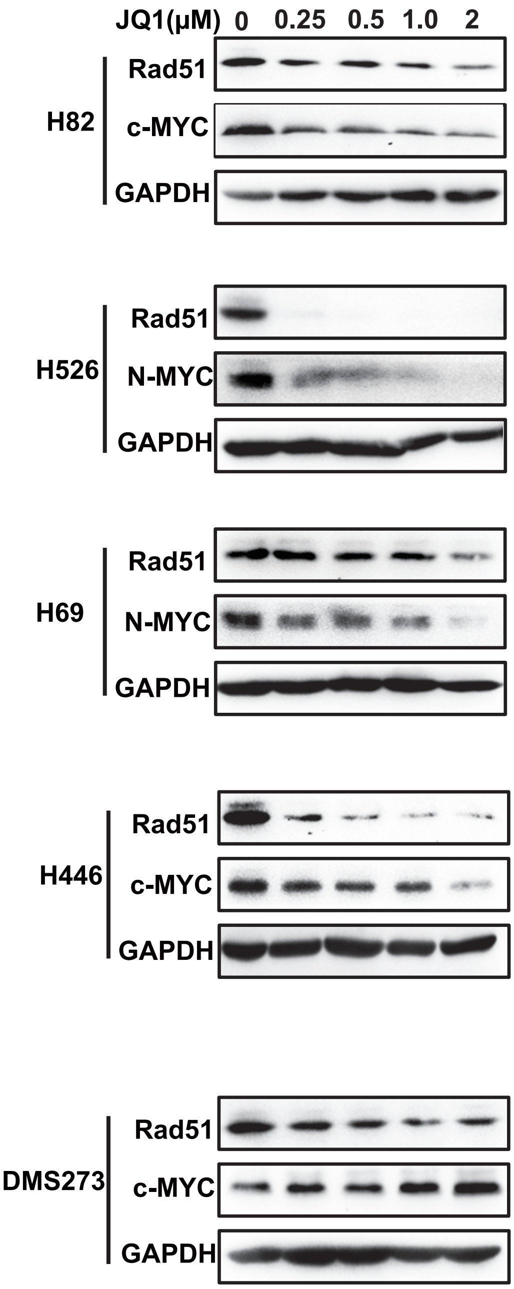
JQ1(μM) 0 0.25 0.5 1.0 2
H82
Rad51
c-MYC
GAPDH
H526
Rad51
N-MYC
GAPDH
H69
Rad51
N-MYC
GAPDH
H446
Rad51
c-MYC
GAPDH
DMS273
Rad51
c-MYC
GAPDH

Figure S5.

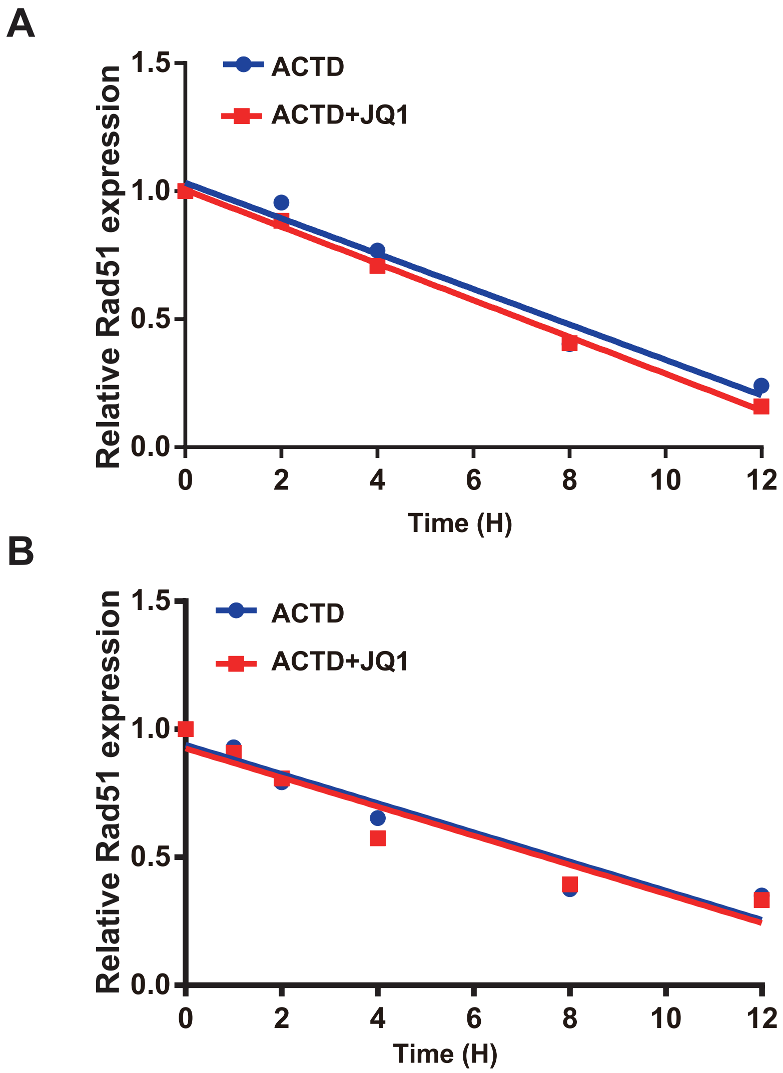
A
ACTD
ACTD+JQ1
Relative Rad51 expression
1.5
1.0
0.5
0.0
0
2
4
6
8
10
12
Time (H)
B
ACTD
ACTD+JQ1
Relative Rad51 expression
1.5
1.0
0.5
0.0
0
2
4
6
8
10
12
Time (H)

Figure S6.

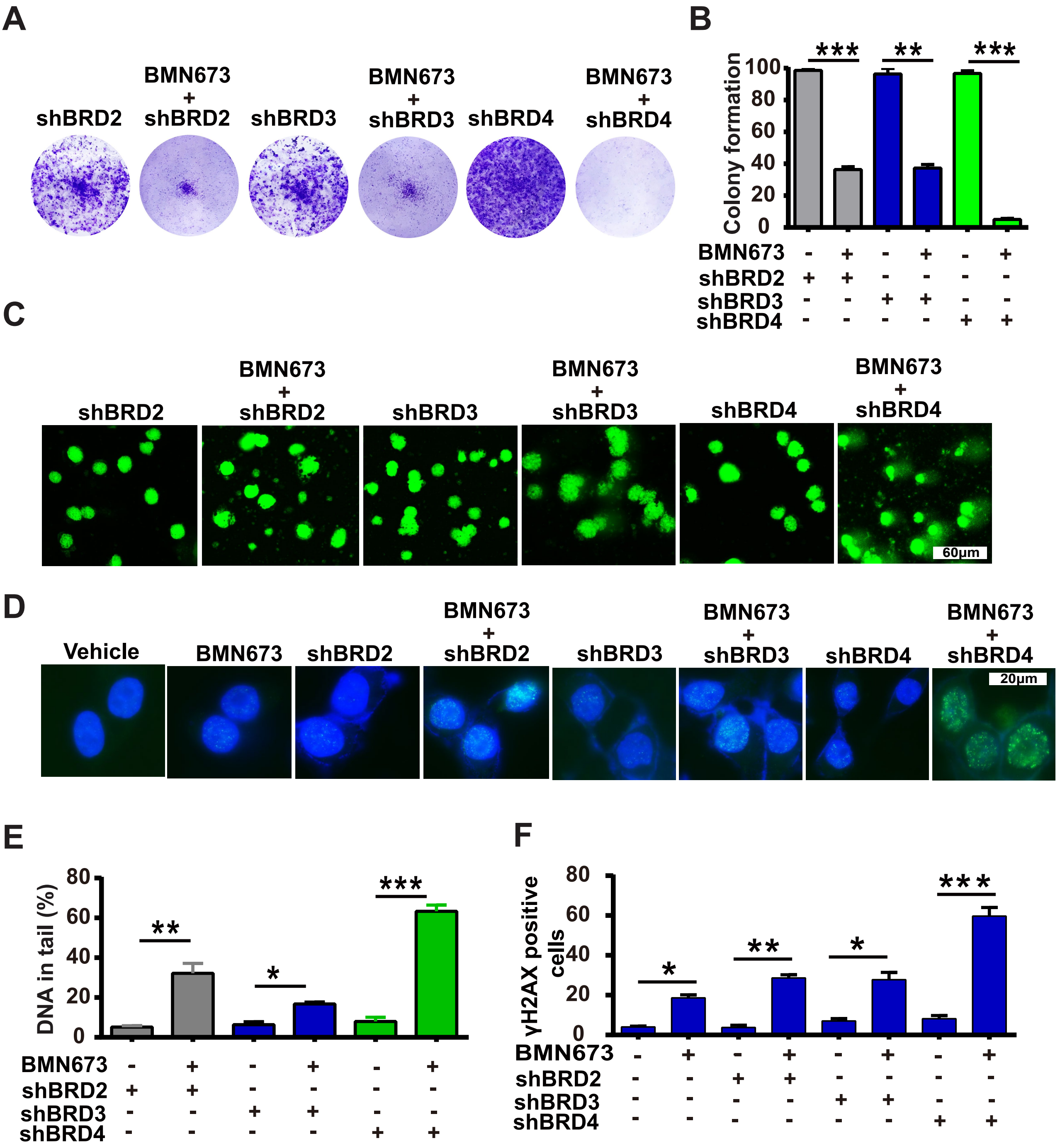
A
BMN673 + shBRD2
shBRD2
shBRD3
BMN673 + shBRD3
shBRD4
BMN673 + shBRD4
B
Colony formation
***
**
***
BMN673
shBRD2
shBRD3
shBRD4
C
60µm
D
Vehicle
BMN673
20µm
E
DNA in tail (%)
*
F
γH2AX positive cells

Figure S7.

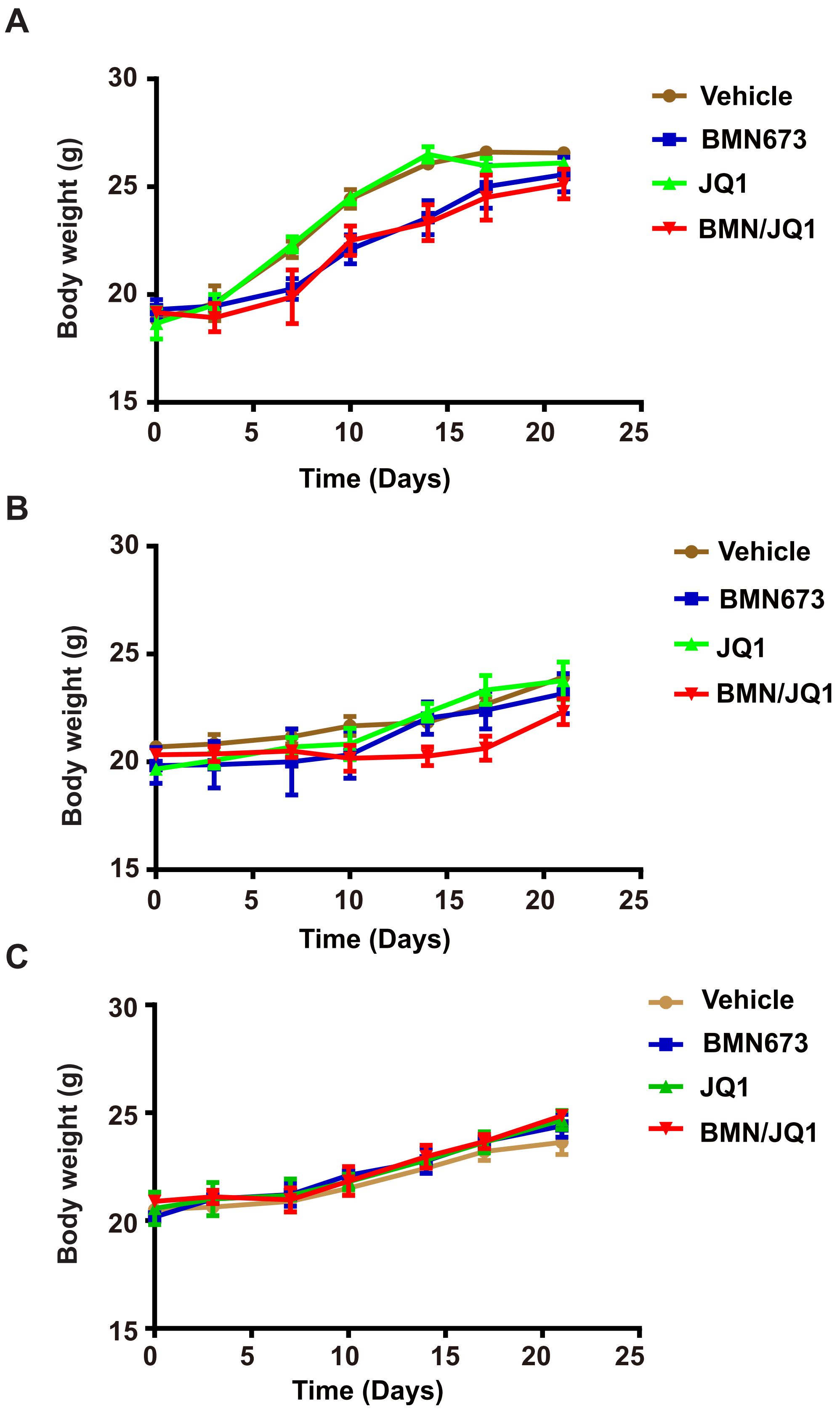
A
Body weight (g)
30
25
20
15
0
5
10
15
20
25
Time (Days)
Vehicle
BMN673
JQ1
BMN/JQ1
B
Body weight (g)
30
25
20
15
0
5
10
15
20
25
Time (Days)
Vehicle
BMN673
JQ1
BMN/JQ1
C
Body weight (g)
30
25
20
15
0
5
10
15
20
25
Time (Days)
Vehicle
BMN673
JQ1
BMN/JQ1

Figure S8.

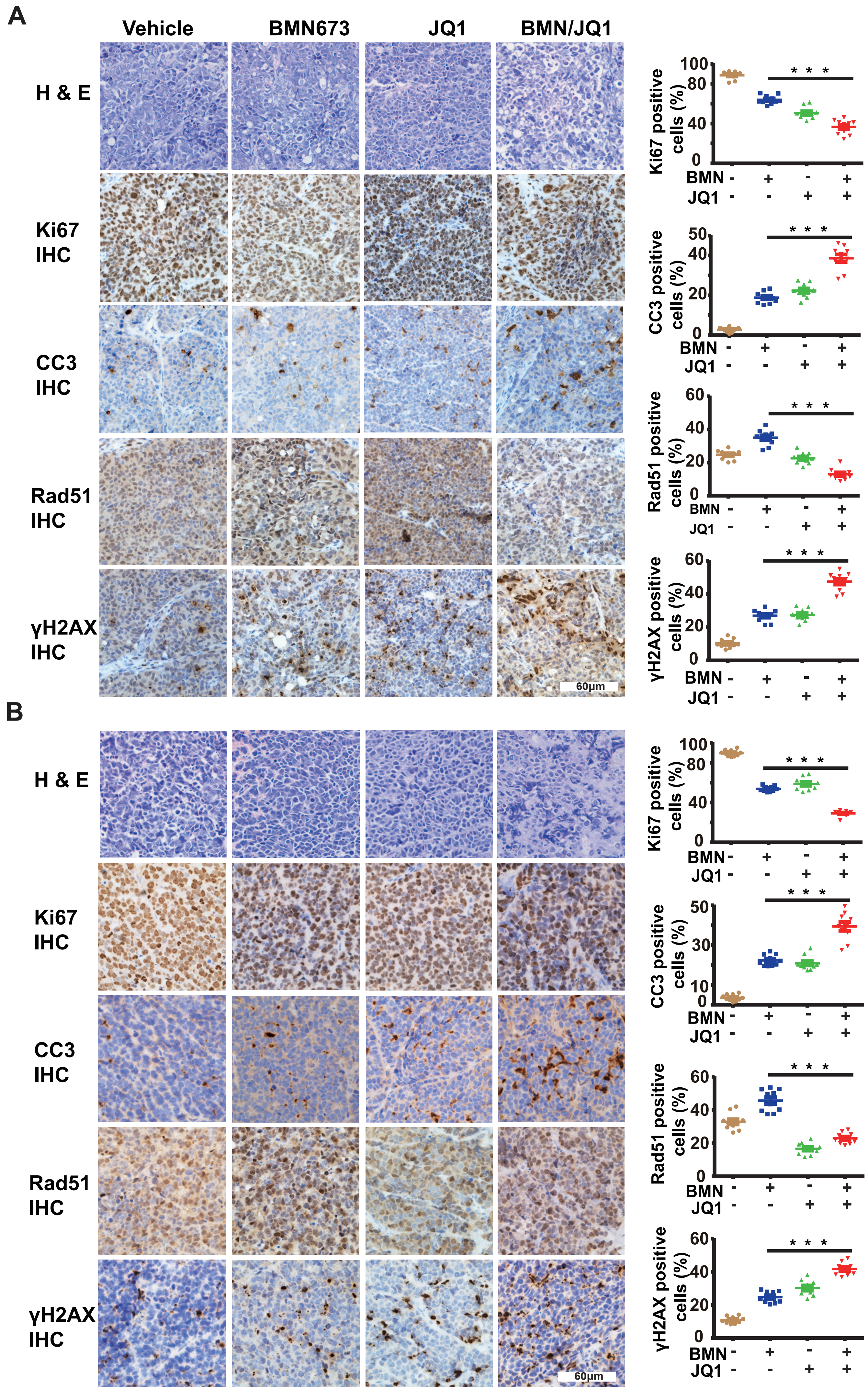

A
Vehicle
BMN673
JQ1
BMN/JQ1
H & E
Ki67 IHC
CC3 IHC
Rad51 IHC
γH2AX IHC
60μm
Ki67 positive cells (%)
CC3 positive cells (%)
Rad51 positive cells (%)
γH2AX positive cells (%)
BMN - + - +
JQ1 - - + +
* * *
B
H & E
Ki67 IHC
CC3 IHC
Rad51 IHC
γH2AX IHC
60μm
Ki67 positive cells (%)
CC3 positive cells (%)
Rad51 positive cells (%)
γH2AX positive cells (%)
BMN - + - +
JQ1 - - + +
* * *

Figure S9.

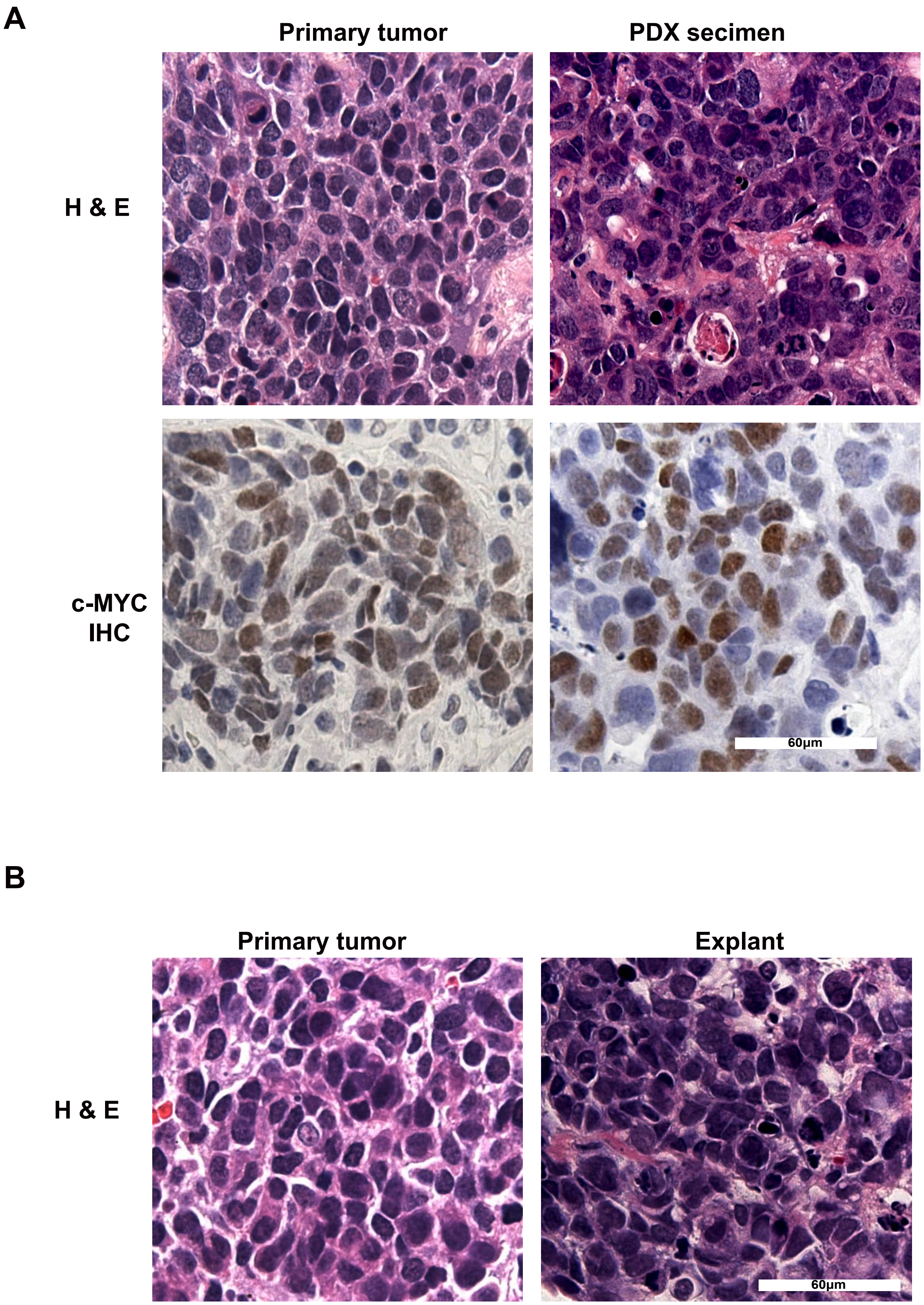

A
Primary tumor
PDX secimen
H & E
c-MYC
IHC
60µm
B
Primary tumor
Explant
H & E
60µm

Figure S10.

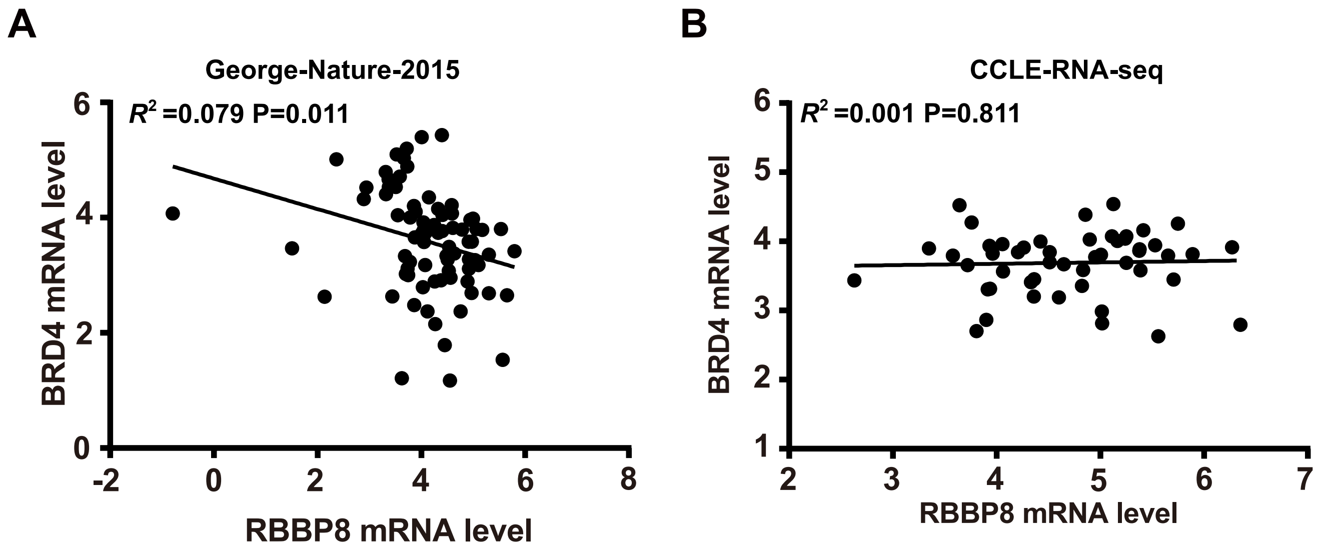
A
George-Nature-2015
R2 =0.079 P=0.011
BRD4 mRNA level
RBBP8 mRNA level
B
CCLE-RNA-seq
R2 =0.001 P=0.811
BRD4 mRNA level
RBBP8 mRNA level

Figure S11.
