# Supplementary material for: The MYC Paralog-PARP1 Axis as a Potential Therapeutic Target in MYC Paralog-Activated Small Cell Lung Cancer
Source: Front Oncol. 2020 Oct 8;10:565820. doi: 10.3389/fonc.2020.565820 (PMC7578565; doi:10.3389/fonc.2020.565820)
Supplement: Supplementary file 2 [file Table_1.docx]

**Supplementary Information**

**Supplementary Figure 1.** DDR-related genes tend to be highly expressed in *MYC* paralog-high SCLC tumor tissues. Scatter plots of the expression of *MYC* paralogs relative to the expression of indicated DDR-related genes in SCLC primary tumors (n=81).

**Supplementary Figure 2.** Effects of JQ1 and BMN673 on growth of SCLC cells. **A-B,** *MYC* paralog-dependent (**A**) and independent (**B**) SCLC cells were treated with BMN673 and JQ1 as single agents or in combination for 72 hours and then subjected to CellTiter-Glo Luminescent assay. The combined drug effect was analyzed using the CI equation and presented with FA combinations.

**Supplementary Figure 3**. BET inhibition inhibits PARPi-induced p-CHK1 in SCLC cells. Representative images of p-CHK1 immunofluorescence staining in SHP77 cells with *c-MYC* overexpression treated with indicated drugs for 24 hours, >5 foci were considered as positive. Error bars represent means ± S.D. Scale bar, 20 μm. Quantification of p-CHK1 fluorescence intensities from three independent experiments was shown as mean ± S.D. These data are representative of three independent experiments. **P* < 0.05; ***P* < 0.01; ****P* < 0.001 (Student’s *t* test).

**Supplementary Figure 4.** Effects of BMN673 and JQ1 on the expression of HR-related genes in SCLC cells. RT-qPCR analysis of *RAD51* (**A**), *BRCA1* (**B**) and *BRCA2* (**C**) expression in *MYC* paralog-dependent SCLC cells treated as indicated drugs for 24 hours. Gene expression was normalized to *β-actin*. Error bars represent mean ± S.D. These data are representative of three independent experiments. **P* < 0.05; ***P* < 0.01; ****P* < 0.001 (Student’s *t* test).

**Supplementary Figure 5.** Effects of JQ1 on c-MYC, MYCN and RAD51 expression in *MYC* paralog-dependent SCLC cells. Western blot analysis of c-MYC, MYCN and RAD51 protein levels in *MYC* paralog-dependent SCLC cells treated with increasing concentrations of JQ1 for 72 hours. β-actin served as a loading control.

**Supplementary Figure 6.** Effect of BET inhibition on *RAD51* mRNA stability in SCLC cells. The half-life of *RAD51* mRNA was measured at indicated time points after treatment with the transcription blocker, actinomycin D (5 M) and JQ1 in H526 cells (**A**) and DMS273 cells (**B**). The relative *RAD51* mRNA abundance was normalized to *β-actin* at the indicated time points.

**Supplementary Figure 7**. Knockdown of BRD4 recapitulates the effects of JQ1. **A,** Representative images of clonogenic assays in BRD2 or BRD3 or BRD4 knockdown cells treated with BMN673 (1μM) for ten days. Fresh media with BMN673 was replaced every three days. **B,** Quantifcation of colony formation from three independent experiments was shown as mean ± S.D. **P* < 0.05; ***P* < 0.01; ****P* < 0.001. **C,** Representative images of comet assays in BRD2 or BRD3 or BRD4 knockdown cells treated with BMN673 (1μM) for 48 hours. DNA in the tail was used to measure DNA damage and assessed by CASP software (CaspLab). Scale bar, 60 μm. **D,** Representative images of immunofluorescent detection of γH2AX foci in BRD2 or BRD3 or BRD4 knockdown cells treated with BMN673 (1μM) for 24 hours, Scale bar, 20 μm. Cells with more than 5 foci were considered as positive. **E-F,** Quantification of DNA damage (**E**) and γH2AX foci (**F**) from three independent experiments was shown as mean ± S.D. These data are representative of three independent experiments. *P < 0.05; **P < 0.01; ***P < 0.001 (Student’s t test).

**Supplementary Figure 8.** Body weight change of mice bearing DMS273 (**A**), H526 (**B**) and H196 (**C**) tumors during 21 days treatments of BMN673 (0.33mg/kg/day, p.o.) and JQ1 (40mg/kg/day, i.p.) as single agents or in combination. Data are shown as mean ± S.E.M.

**Supplementary Figure 9**. Combined use of BMN673 and JQ1 is effective *in vivo*. **A-B,** Representative images of histological and immunohistochemical analysis in tumors isolated from DMS273 (**A**) and H526 (**B**) xenograft mice administered with BMN673 and JQ1 alone or in combination for 3 days. Scale bar, 60 μm. Data are shown as mean ± S.E.M. **P* < 0.05; ***P* < 0.01; ****P* < 0.001. H&E, hematoxylin and eosin. CC3, cleaved-caspase3.

**Supplementary Figure 10.** Representative images of H&E and c-MYC immunohistochemistry staining in primary tumor and PDX specimen. **A,** Representative images of H&E staining and c-MYC immunohistochemistry staining in primary tumor and PDX specimen. Scale bar, 60 μm. **B,** Representative images of H&E staining in PDX specimen and tumor explant.

**Supplementary Figure 11.** BRD4 expression was not positively correlated with RBBP8 expression in SCLC. **A-B,** Relative mRNA levels of BRD4 and RBBP8 were plotted in log2 scale in SCLC patient tissues (n=81) (**A**) and SCLC cell lines (n=50) (**B**).

**Supplementary table 1. Primer Sequences for ChIP-PCR. P1: PARP1, R51: RAD51, BS= binding site.**

| Gene name | Primer name | Primer sequence | Bases |
| --- | --- | --- | --- |
| PARP1 | P1BS1-Forward | ACCACAGCCTCAACCTTCAG | 20 |
|  | P1BS1-Reverse | TGAGCCCAGGAGTTCAAGAC | 20 |
|  | P1BS2-Forward | ACAACAAGATCTAGGCCAGGT | 21 |
|  | P1BS2-Reverse | GTGTGTGTGTGTGTGTGTGT | 20 |
|  | P1BS3-Forward | AAGTCGAGGTGGGAGGATTG | 20 |
|  | P1BS3-Reverse | TCGAAATTGTGGTAATGACTGCA | 23 |
|  | P1BS4-Forward | AGAACAATCAAAGGGGTGGCG | 21 |
|  | P1BS4-Reverse | GCCGTTCCCTGATAGATTGC | 20 |
| RAD51 | R51BS1-Forward | ATGCATGCCGGGAGATGTAGT | 21 |
|  | R51BS1-Reverse | TTCGAGGCTAACCACGGCAA | 20 |
|  | R51BS2-Forward | AGATCTCGGTTGGCTGCAACCT | 22 |
|  | R51BS2-Reverse | CAGGGGTTGGAGATAAACCT | 20 |
|  | R51BS3-Forward | CAAAGAGCTGGGATTACAGGCA | 22 |
|  | R51BS3-Reverse | AGCCTGTAATCCCAGAACTTT | 21 |
|  | R51BS4-Forward | TTGGCGGGAATTCTGAAAGC | 20 |
|  | R51BS4-Reverse | ACGCTCCACTTCTCTACTCG | 20 |

**Supplementary table 2. Primer Sequences for qRT-PCR**

| Gene name | Primer name | Primer sequence | Bases |
| --- | --- | --- | --- |
| RAD51 | Rad51- Forward | CAACCCATTTCACGGTTAGAGC | 22 |
|  | Rad51- Reverse | TTCTTTGGCGCATAGGCAACA | 21 |
| BRCA1 | BRCA1- Forward | ACCTTGGAACTGTGAGAACTCT | 22 |
|  | BRCA1- Reverse | TCTTGATCTCCCACACTGCAATA | 23 |
| BRCA2 | BRCA2- Forward | ACAAGCAACCCAAGTGTCAAT | 21 |
|  | BRCA2- Reverse | TGAAGCTACCTCCAAAACTGTG | 22 |

**Supplementary table 3. DNA oligonucleotides used for shRNA studies**

| genes | shRNA targeting sequences |
| --- | --- |
| BRD2#1 | GCTGCCCTATTCACTTCTAAG |
| BRD2#2 | CCGGAAGCCCTACACCATTAA |
| BRD3#1 | CCCAAGAGGAAGTTGAATTAT |
| BRD3#2 | CCAAGGAAATGTCTCGGATAT |
| BRD4#1 | GCCAAATGTCTACACAGTATA |
| BRD4#2 | TGAACCTCCCTGATTACTATA |
| BRD4#3 | ATTGGACACGGACTCTTAATA |

**Supplementary table 4. siRNA Sequence**

| siRNA name | Target sequence |
| --- | --- |
| Hs_c-MYC_1 | GCTTGTACCTGCAGGATCT |
| Hs_c-MYC_2 | GAGGATATCTGGAAGAAAT |
| Hs_c-MYC_3 | GGAAGAAATCGATGTTGTT |

**Supplementary table 5. DNA oligonucleotides used to clone cDNAs**

| cDNA | Primers |
| --- | --- |
| Hs_c-MYC | Forward 5’CGGGATCCCGCTGGATTTTTTTCGGGTAG3’ |
|  | Reverse 5’ACGCGTCGACTTACGCACAAGAGTTCCG3’ |

**Supplementary table 6. The status of *MYC* paralogs in SCLC cell lines**

| **SCLC cell lines** | **MYC** | **MYCN** | **MYCL** |
| --- | --- | --- | --- |
| H82 | Amp |  |  |
| H526 |  | Amp |  |
| H69 |  | Amp |  |
| H446 | Amp |  |  |
| DMS273 | Amp |  | Amp |
| DMS53 | Overexpression |  |  |
| SHP77 |  |  |  |
| H1963 |  |  | Amp |
| H196 |  |  |  |
| H2066 |  |  |  |

Amp: Amplification
